# Spatiotemporal prediction of infectious diseases using structured Gaussian processes with application to Crimean–Congo hemorrhagic fever

**DOI:** 10.1371/journal.pntd.0006737

**Published:** 2018-08-17

**Authors:** Çiğdem Ak, Önder Ergönül, İrfan Şencan, Mehmet Ali Torunoğlu, Mehmet Gönen

**Affiliations:** 1 Graduate School of Sciences and Engineering, Koç University, İstanbul, Turkey; 2 Department of Infectious Diseases and Clinical Microbiology, School of Medicine, Koç University, İstanbul, Turkey; 3 Public Health Directorate, Ministry of Health, Ankara, Turkey; 4 Department of Industrial Engineering, College of Engineering, Koç University, İstanbul, Turkey; 5 School of Medicine, Koç University, İstanbul, Turkey; Oxford University Clinical Research Unit, VIETNAM

## Abstract

**Background:**

Infectious diseases are one of the primary healthcare problems worldwide, leading to millions of deaths annually. To develop effective control and prevention strategies, we need reliable computational tools to understand disease dynamics and to predict future cases. These computational tools can be used by policy makers to make more informed decisions.

**Methodology/Principal findings:**

In this study, we developed a computational framework based on Gaussian processes to perform spatiotemporal prediction of infectious diseases and exploited the special structure of similarity matrices in our formulation to obtain a very efficient implementation. We then tested our framework on the problem of modeling Crimean–Congo hemorrhagic fever cases between years 2004 and 2015 in Turkey.

**Conclusions/Significance:**

We showed that our Gaussian process formulation obtained better results than two frequently used standard machine learning algorithms (i.e., random forests and boosted regression trees) under temporal, spatial, and spatiotemporal prediction scenarios. These results showed that our framework has the potential to make an important contribution to public health policy makers.

## Author summary

Infectious diseases cause important health problems worldwide and create difficult challenges for public health policy makers. That is why they need reliable computational tools to better understand disease and to predict case counts. They will benefit from such computational tools to make more informed decisions in developing control and prevention strategies. We formulated a computational framework that can be used to model spatial, temporal, or spatiotemporal dynamics of infectious diseases. We showed the utility of our framework on the problem of modeling Crimean–Congo hemorrhagic fever in Turkey.

## Introduction

Infectious diseases constitute a major part of healthcare burden worldwide, leading to millions of deaths annually, which are especially seen among poor and young populations in low and middle income countries [1]. In addition to pandemic infectious diseases such as influenza and tuberculosis, there are also emerging infectious diseases such as Ebola virus disease and Zika fever, which require a worldwide effort to combat. Thus, predicting the case counts of infectious diseases is of great importance in developing control and prevention strategies. In particular, there might be spatial dependencies (e.g., humid conditions for malaria) and temporal dependencies (e.g., seasonal effects for influenza) that control the emergence and spread of such diseases [2].

To be able to develop protective measures against infectious diseases, it is very important (i) to clearly identify the disease spread and (ii) to make reliable predictions for future cases. When the disease spread is known, policy makers can develop preventive strategies against, for instance, environmental factors that promote the disease. Once we have reliable predictions for future cases, policy makers can make informed decisions on, for example, vaccine purchases, public awareness campaigns and training programs for healthcare workers.

Machine learning algorithms can contribute to the control of infectious diseases by addressing aforementioned two aims. In the literature, standard machine learning algorithms such as random forests [3] and boosted regression trees [4, 5] were frequently used in ecological and epidemiological applications [6–10]. These algorithms have been picked by the applied researchers mainly because they have a relatively simple interface for nonspecialists. However, they might fail to capture highly complex dependencies in disease modeling scenarios. Thus, we used Gaussian processes [11] to be able to identify highly nonlinear dependencies and to make more reliable predictions.

We proposed a computational framework that uses Gaussian processes as the basic building block to perform spatiotemporal prediction of infectious diseases. We first noted that the kernel matrices have a special structure owing to their dependencies on both spatial and temporal covariates and then exploited this special structure to obtain a very efficient inference algorithm. We tested our proposed framework on Turkey’s country-wide surveillance data set of a vector-borne infectious disease Crimean–Congo hemorrhagic fever, which is a widespread endemic infectious disease seen in Africa, the Balkans, the Middle East, and Asia with a case fatality rate of 5–40% [12].

**Fig 1:**
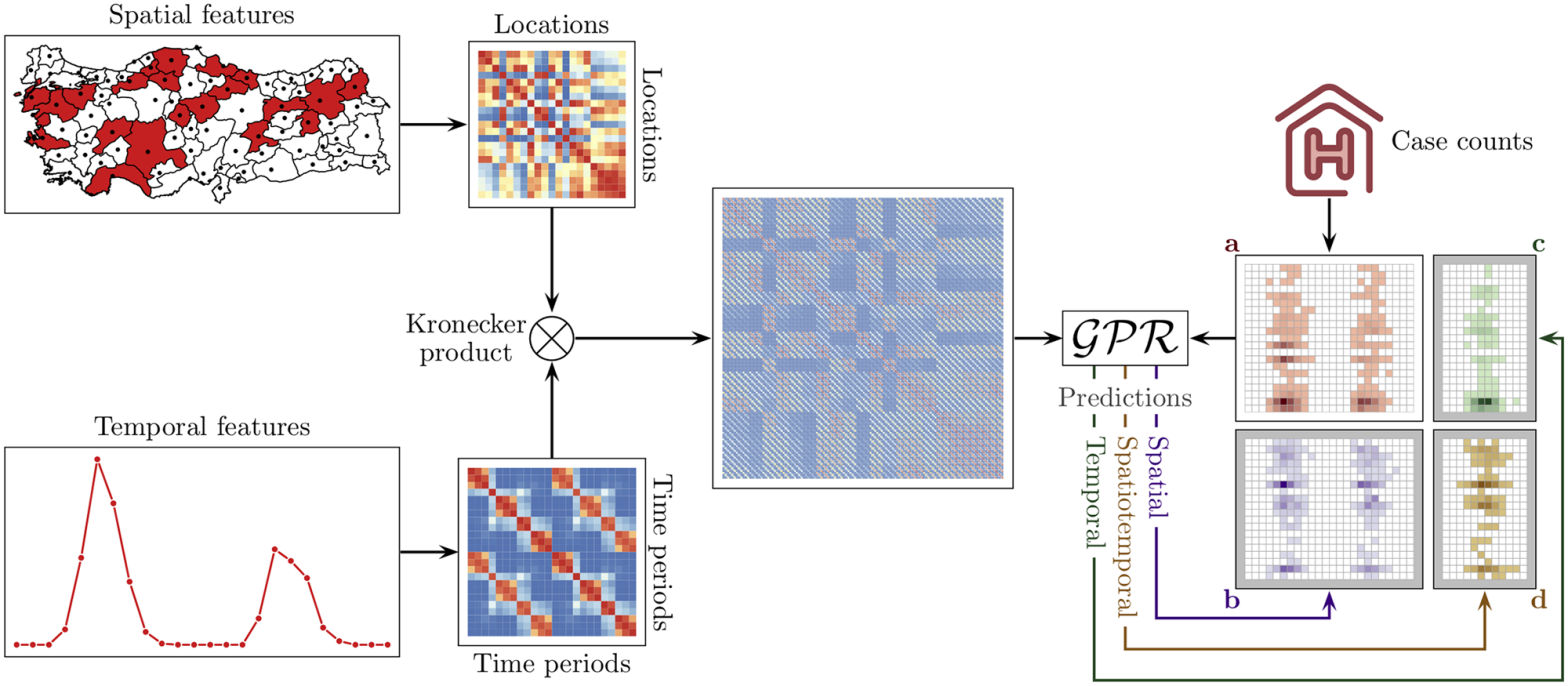
Overview of our proposed computational framework to perform spatiotemporal prediction of infectious diseases. (**a**) Reported case counts are given for location and time period pairs. The proposed framework can be used for three different prediction scenarios: (**b**) spatial prediction, (**c**) temporal prediction, and (**d**) spatiotemporal prediction.

We present the overview of our proposed computational framework with three possible prediction scenarios in Fig 1. We assume that the reported case counts of location and time period pairs have been recorded with additional information about their spatial and temporal properties. We first extract spatial and temporal features for each location and time period, respectively, from these properties. We then calculate two similarity matrices among locations and time periods, respectively, using the extracted features. These two similarity matrices are combined to obtain a larger similarity matrix between location and time period pairs. Using the combined similarity matrix and reported cases counts, we train a Gaussian process regression model to be able to make predictions under three different scenarios: (i) temporal prediction (i.e., predicting case counts for future time periods, leading to predicting disease prevalence for each location in the future), (ii) spatial prediction (i.e., predicting case counts for unseen locations, leading to predicting disease spread within the same time frame in other locations), which can be used to complete missing case counts for the locations that we could not obtain historical data, and (iii) spatiotemporal prediction (i.e., predicting case counts for unseen location and future time period pairs, leading to predicting disease spread to new locations in the future), which is especially important to be able to prepare against emerging infectious diseases since there will be no historical data for the locations that experience the disease for the first time.

## Materials and methods

In this study, we proposed a computational framework to perform spatiotemporal prediction of infectious diseases. To test this framework, we addressed an important public health problem in Turkey, namely, Crimean–Congo hemorrhagic fever (CCHF), which is a vector-borne infectious disease transmitted by infected tick bites and exposure to blood or bodily fluids of the infected cases.

### Materials

We used an unpublished surveillance data set of 9,636 CCHF infection cases reported in Turkey between years 2004 and 2015, which was collected by the Ministry of Health of Turkey (S1 File). The reported cases were mainly because of infected tick bites, and they were diagnosed with clinical symptoms such as fever, myalgia, and bleeding from various sites. These infected cases were also confirmed with blood tests.

The Ministry of Health of Turkey provided us with spatial information (province, district, and town names) and temporal information (year and month) for each case, which made this data set suitable for studying spatiotemporal characteristics of CCHF. The data set does not include clinical covariates of infected cases, which forces our study to investigate only spatial and temporal covariates.

#### Spatial covariates

**Fig 2:**
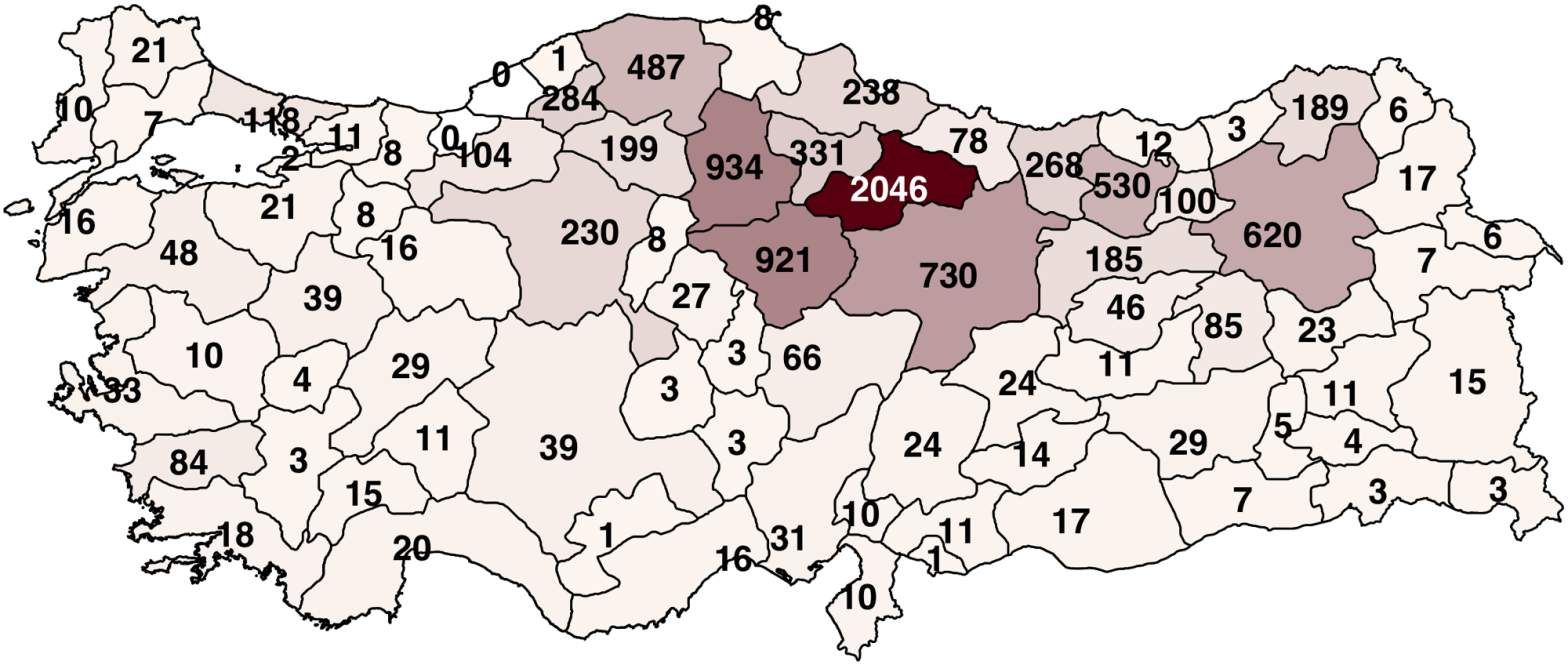
The total numbers of infected cases reported in 81 provinces of Turkey between years 2004 and 2015. Note that the northern and northeastern regions had strikingly high numbers of infected cases. The numbers were shown on the province centers. This map was generated using the Turkish administrative map downloaded from https://www.gadm.org and the R package maps version 3.3.0 at https://cran.r-project.org/web/packages/maps.

We used the infected case counts of provinces to capture the spatial spread of CCHF since finer resolutions such as district or town level gives us very sparse case counts. Fig 2 shows the total numbers of infected cases reported in 81 provinces of Turkey between years 2004 and 2015, whereas annual numbers of infected cases can be seen in S1, S2, S3, S4, S5, S6, S7, S8, S9, S10, S11 and S12 Figs. CCHF cases had mainly been observed in northern and northeastern regions of Turkey (e.g., 2,046 of 9,636 infected cases were reported in a single northern province), and other regions had strikingly fewer infected cases (e.g., southern provinces had one to three infected cases per year). This confirmed that CCHF has a strong spatial dependency, which was reported by several earlier studies [13–15], owing to mainly spatial differences in wild-life and livestock animal populations carrying ticks. We extracted latitude and longitude coordinates of each province centre, leading to two spatial covariates.

#### Temporal covariates

**Fig 3:**
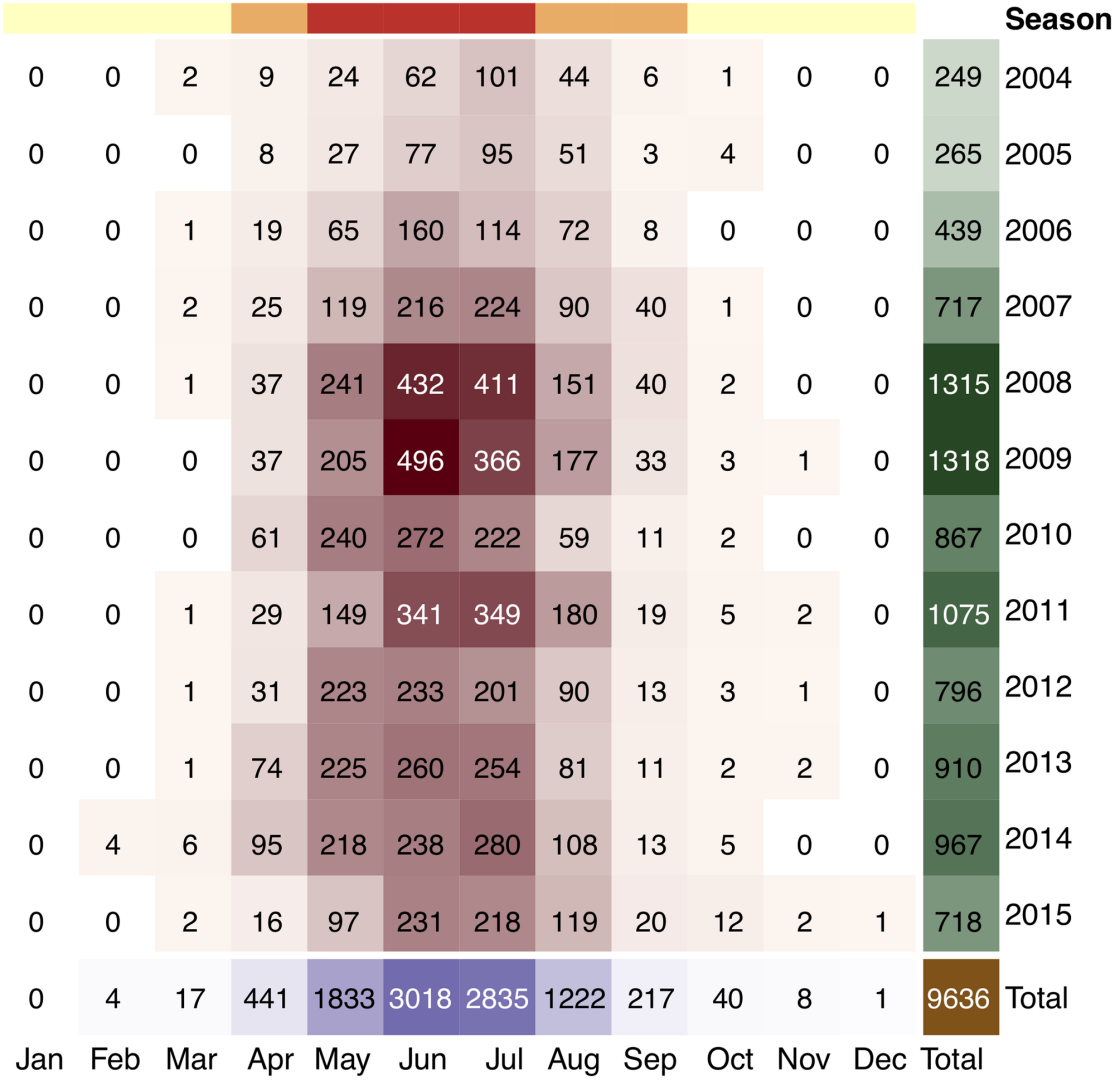
The numbers of country-wide infected cases for each month between years 2004 and 2015. The total numbers of infected cases for each month and each year were also reported as column and row sums, respectively. The columns were annotated by their seasonal group information at the top (yellow: cold; orange: warm; red: hot). Note that there is an annual periodicity of cases and a striking seasonal variation over infected cases.

We used the monthly infected case counts since we did not have data for finer resolutions and ticks become dormant (i.e., inactive) during cold weather, which makes periods longer than month unable to capture the temporal dynamics of CCHF. Fig 3 shows the numbers of country-wide infected cases for each month between years 2004 and 2015. CCHF cases had been observed frequently during hot months (e.g., May, June, and July), moderately during warm months (e.g., April, August, and September) and rarely during cold months (e.g., October, November, December, January, February, and March). This confirmed that CCHF has a strong temporal dependency, which was again reported by several earlier studies [16–18], owing to mainly life or sleep cycles of ticks. We encoded each time period by three temporal covariates: the year, month, and seasonal group (i.e., hot, warm, or cold) it belongs to.

### Methods

Infectious disease spread is usually driven by both location and time, which means nearby locations and time periods have similar characteristics. The disease spreads to adjacent province much more easily than distant provinces due to spatial dependency. Case counts in consecutive time periods or in time periods within the same season are usually heavily correlated due to temporal dependency.

We suggest using Gaussian process regression (GPR), which is suitable to capture highly complex dependencies between input and output variables thanks to its nonlinear nature brought by kernel functions. We propose a computational strategy based on GPR that enables us to perform predictions under spatial (i.e., predicting case counts for unseen locations), temporal (i.e., predicting case counts for future time periods) and spatiotemporal scenarios (i.e., predicting counts for unseen location and future time period pairs) for infectious diseases.

We first give a brief description of GPR. We then show how GPR can be modified for infectious disease modeling by introducing a structured kernel function based on two separate kernel functions over spatial and temporal covariates, respectively, and how this modified GPR formulation can be implemented very efficiently. We describe three different prediction scenarios encountered in spatiotemporal modeling of infectious diseases. We lastly discuss two baseline algorithms from the literature that will be used to benchmark against.

#### Gaussian process regression

Gaussian processes have been used in many applications for temporal and spatial prediction such as environmental surveillance [19], reconstruction of sea surface temperatures [20], drug–target interaction prediction [21], global land-surface precipitation prediction [22], and wind power forecasting [23] as well as spatiotemporal modeling [24, 25]. There is also a significant number of studies on Gaussian processes with application to epidemiology [26–29].

For a given training data set $\{(x_{i},y_{i}){\}}_{i=1}^{N}$, GPR uses a probabilistic formulation to model the relationship between the input covariates and the output as follows [11]:

$$\begin{array}{cc} y & =f+\xi ,\\ f|X & ∼\text{Normal}(f;0,K),\\ \xi |\sigma_{y}^{2} & ∼\text{Normal}(\xi ;0,\sigma_{y}^{2}I), \end{array}$$

where ***y*** = [*y*_1_ *y*_2_ ⋯ *y*_*N*_]^⊤^ is the vector of observed output values, ***f*** = [*f*_1_ *f*_2_ ⋯ *f*_*N*_]^⊤^ is the vector of underlying true output values for the corresponding input data instances **X** = [***x***_1_ ***x***_2_ ⋯ ***x***_*N*_], ***ξ*** = [*ξ*_1_ *ξ*_2_ ⋯ *ξ*_*N*_]^⊤^ is the vector of measurement noise values that are assumed to follow an isotropic multivariate normal distribution with the variance parameter $\sigma_{y}^{2}$, and **0** and **I** are the vector of zeros and the identity matrix of proper sizes, respectively.

The true output values ***f*** are assumed to follow a multivariate normal distribution with the mean **0** and the covariance **K** defined as

$$\begin{array}{c} K=[\begin{array}{cccc} k(x_{1},x_{1}) & k(x_{2},x_{1}) & \cdots & k(x_{N},x_{1})\\ k(x_{1},x_{2}) & k(x_{2},x_{2}) & \cdots & k(x_{N},x_{2})\\ \vdots & \vdots & \ddots & \vdots \\ k(x_{1},x_{N}) & k(x_{2},x_{N}) & \cdots & k(x_{N},x_{N}) \end{array}], \end{array}$$

where *k*(⋅,⋅) is a kernel function that calculates a similarity measure between two data instances. By integrating out the true output values ***f***, it can be shown that the observed output values ***y*** have the following form:

$$\begin{array}{c} y|X,\sigma_{y}^{2}∼\text{Normal}(y;0,K+\sigma_{y}^{2}I), \end{array}$$

where we can use the properties of the multivariate normal distribution to find the predictive distribution of an unknown output value *y*_⋆_ for an unseen data instance ***x***_⋆_. We first write the joint distribution of (***y***, *y*_⋆_) and then find the conditional distribution of *y*_⋆_ to obtain its predictive distribution, which is also a multivariate normal distribution with the following mean and variance:

$$\begin{array}{c} E[y_{⋆}|x_{⋆},X,y,\sigma_{y}^{2}]=k_{⋆}^{⊤}{\left(K+\sigma_{y}^{2}I\right)}^{-1}y, \end{array}$$ (1)

$$\begin{array}{c} \text{Var}[y_{⋆}\left|x_{⋆},X,y,\sigma_{y}^{2}\right]=k\left(x_{⋆},x_{⋆}\right)-k_{⋆}^{⊤}{\left(K+\sigma_{y}^{2}I\right)}^{-1}k_{⋆}, \end{array}$$ (2)

where ***k***_⋆_ = [*k*(***x***_⋆_, ***x***_1_) *k*(***x***_⋆_, ***x***_2_) ⋯ *k*(***x***_⋆_, ***x***_*N*_)]^⊤^.

#### Structured GPR

For large data sets, Gaussian processes might become computationally intensive. Several decomposition algorithms have been previously proposed to make the inference faster such as Nyström approximation [11], approximation using Hadamard and diagonal matrices [30], or Kronecker methods [21, 31–36].

In spatiotemporal modeling, we can represent each data instance ***x***_*i*_ as a pair of location and time period vectors (***s***_*l*_, ***t***_*p*_), where *l* indexes locations, *p* indexes time periods, *L* is the number of locations, and *P* is the number of time periods. We can also form a response matrix **Y** of size *L* × *P* to store *y*_*i*_ values of these pairs.

In this case, the kernel function between data instances can be written as the multiplication of two separate kernel functions:

$$\begin{array}{c} k\left(x_{i},x_{j}\right)=k\left(\left(s_{l},t_{p}\right),\left(s_{m},t_{q}\right)\right)=k_{s}\left(s_{l},s_{m}\right)k_{t}\left(t_{p},t_{q}\right), \end{array}$$

where *k*_*s*_(⋅,⋅) gives the similarity between geographical locations using spatial features, and *k*_*t*_(⋅,⋅) calculates the similarity between time periods using temporal features.

The kernel matrix calculated on the training instances can be written as the Kronecker product of two smaller kernel matrices calculated on the geographical locations and the time periods, respectively.

$$\begin{array}{c} K=K_{s}⊗K_{t}, \end{array}$$

where **K**, **K**_*s*_, and **K**_*t*_ are of sizes *LP* × *LP*, *L* × *L*, and *P* × *P*, respectively. Similarly, the vector that stores kernel function outputs between the test instance and the training instances can be written as

$$\begin{array}{c} k_{⋆}=k_{s,⋆}⊗k_{t,⋆}. \end{array}$$

We can update the mean prediction equation of standard Gaussian process in Eq (1) with the Kronecker kernel:

$$\begin{array}{c} E[y_{⋆}|x_{⋆},X,Y,\sigma_{y}^{2}]={\left(k_{s,⋆}⊗k_{t,⋆}\right)}^{⊤}{\left(K_{s}⊗K_{t}+\sigma_{y}^{2}I\right)}^{-1}\mathrm{vec}\left(Y\right), \end{array}$$ (3)

where vec(⋅) converts the input matrix into a column vector. The variance prediction equation in Eq (2) can also be updated as

$$\begin{array}{c} \text{Var}[y_{⋆}\left|x_{⋆},X,Y,\sigma_{y}^{2}\right]=k_{s}\left(s_{⋆},s_{⋆}\right)k_{t}\left(t_{⋆},t_{⋆}\right)\\ \;-{\left(k_{s,⋆}⊗k_{t,⋆}\right)}^{⊤}{\left(K_{s}⊗K_{t}+\sigma_{y}^{2}I\right)}^{-1}\left(k_{s,⋆}⊗k_{t,⋆}\right). \end{array}$$ (4)

**Implementation details.** The matrix inversion operation in Eqs (3) and (4) is computationally expensive since it inverts an *LP* × *LP* matrix. To benefit from the special structure of our kernel matrices, we will use the properties of the Kronecker product as described in [37]. First, we factorize the smaller kernel matrices **K**_*s*_ and **K**_*t*_ using singular value decomposition:

$$\begin{array}{c} K_{s}=U_{s}D_{s}U_{s}^{⊤},\\ K_{t}=U_{t}D_{t}U_{t}^{⊤}, \end{array}$$

where the left-singular vectors and right-singular vectors are identical since the kernel matrices are positive semi-definite.

We then write the Kronecker product of the spatial and temporal kernel matrices using the singular values and singular vectors of each matrix:

$$\begin{array}{c} K_{s}⊗K_{t}=\left(U_{s}⊗U_{t}\right)\left(D_{s}⊗D_{t}\right){\left(U_{s}⊗U_{t}\right)}^{⊤}. \end{array}$$

The matrix inversion operation can be replaced by the following formula:

$$\begin{array}{c} {\left(K_{s}⊗K_{t}+\sigma_{y}^{2}I\right)}^{-1}=\left(U_{s}⊗U_{t}\right){\left(D_{s}⊗D_{t}+\sigma_{y}^{2}I\right)}^{-1}{\left(U_{s}⊗U_{t}\right)}^{⊤}. \end{array}$$ (5)

We can rewrite the mean and variance predictions in Eqs (3) and (4) using the Kronecker inversion rule in Eq (5). After this change, these two equations can be calculated very efficiently using Kronecker matrix-vector multiplications and by inverting a diagonal matrix.

**Fig 4:**
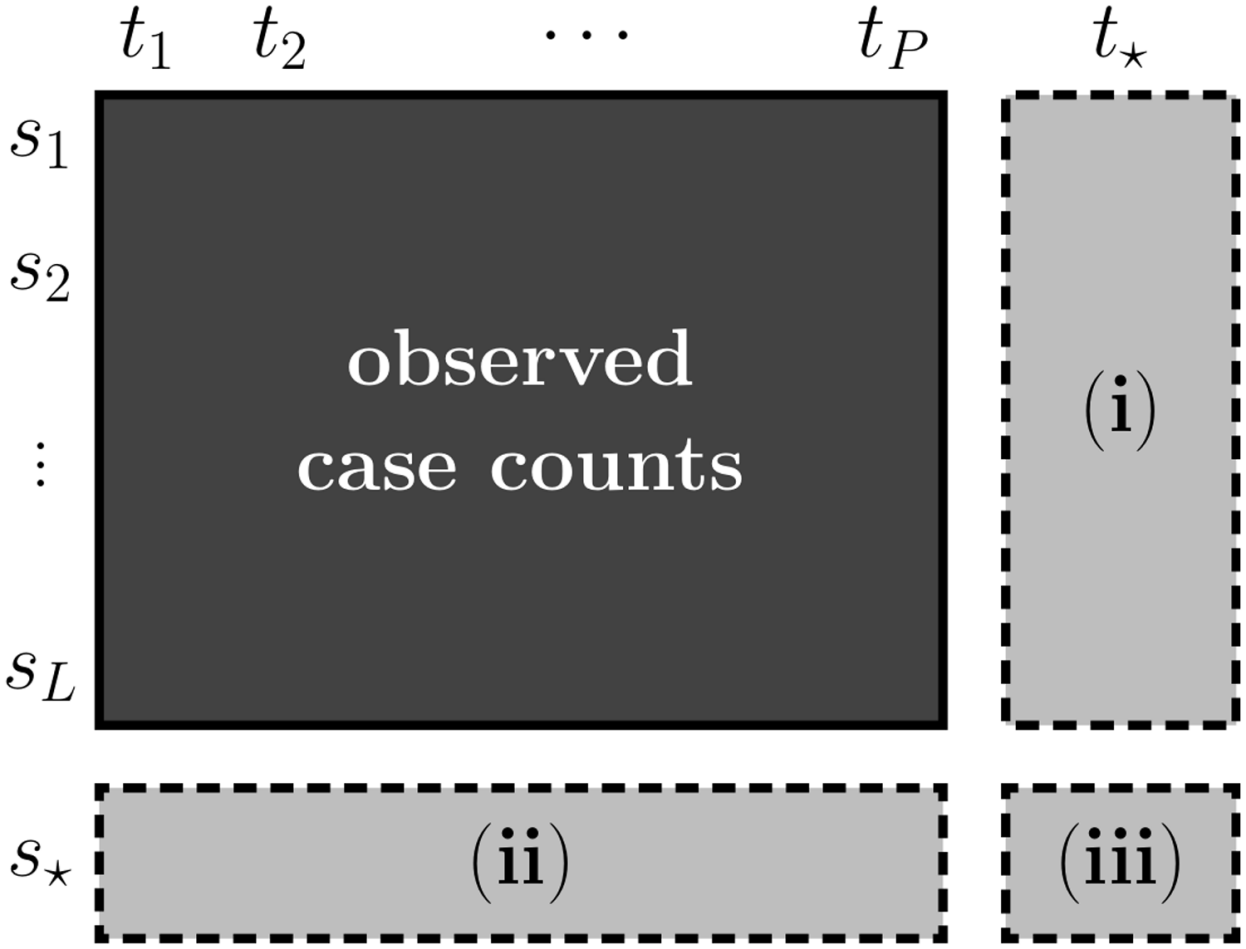
Three prediction scenarios. (i) temporal scenario to predict case counts of future time points on the training locations, (ii) spatial scenario to predict case counts of unseen locations at the training time points, and (iii) spatiotemporal scenario to predict case counts of unseen locations at future time points.

**Infectious disease modeling using structured GPR.** In this study, we use structured GPR formulation to predict case counts under three different scenarios (Fig 4): (i) predicting case counts for a future time period *t*_⋆_, (ii) predicting case counts for an unseen location *s*_⋆_, and (iii) predicting case counts for an unseen location and future time period pair (*s*_⋆_, *t*_⋆_). In all scenarios, we assume that we are given case counts within a list of locations for a number of time periods.

**Predicting case counts for a future time period.** In the first scenario, we are interested in finding case counts in the observed locations for a future time period. This amounts to making predictions for (***s***_*l*_, ***t***_⋆_) pairs, where ***s***_*l*_ is one of the locations in our training set.

**Predicting case counts for an unseen location.** In the second scenario, we are interested in finding case counts in an unseen location for the observed time periods. This amounts to making predictions for (***s***_⋆_, ***t***_*p*_) pairs, where ***t***_*p*_ is one of the time periods in our training set.

**Predicting case counts for an unseen location and future time period pair.** In the third scenario, we are interested in finding case counts in an unseen location for a future time period. This amounts to making predictions for (***s***_⋆_, ***t***_⋆_) pairs.

**Baseline algorithms.** Several off-the-shelf machine learning algorithms can be used to perform spatiotemporal prediction of infectious diseases. In this study, we compared our method against two particular baseline algorithms, namely, random forests regression (RFR) and boosted regression trees (BRT). We have two main reasons for these particular choices: (i) Both RFR and BRT are frequently used and considered as the standard machine learning algorithms to capture temporal, spatial, and spatiotemporal dependencies in ecological and epidemiological applications [6–10]. (ii) Both RFR and BRT are nonlinear algorithms as our structured GPR formulation.

**Random forests regression.** RFR algorithm combines several regression trees trained on different portions of the input covariates [3]. As a result, the obtained regression trees give diverse decision rules, and combining several trees produces more robust results.

**Boosted regression trees.** BRT algorithm is based on the idea of combining weak learners to obtain better learners (i.e., boosting) and uses decision trees trained on different subsamples of training instances as weak learners [4, 5].

#### Experimental settings and performance metrics

We created three scenarios to perform experiments for temporal, spatial, and spatiotemporal prediction.

For temporal prediction, we took the first 10 years and the remaining two years as training and test sets, respectively. We first trained the three algorithms using case counts of 81 provinces over 10 years (120 months) as the observed response matrix, leading to a training set of 9,720 instances (81 provinces × 120 months). We then tested the trained models by predicting observed case counts of 81 provinces for the remaining two years (24 months), leading to a test set of 1,944 instances (81 provinces × 24 months).

For spatial prediction, we divided 81 provinces into two groups by first ordering their total case counts and then taking odd- and even-numbered provinces as training and test sets, respectively (S13 Fig). We first trained the three algorithms using case counts of 41 training provinces over 12 years (144 months) as the observed response matrix, leading to a training set of 5,904 instances (41 provinces × 144 months). We then tested the trained models by predicting observed case counts of 40 test provinces for the same time periods, leading to a test set of 5,760 instances (40 provinces × 144 months).

For spatiotemporal prediction, we took the intersection of training sets (respectively, test sets) of the first two scenarios as the training set (respectively, test set). We first trained the three algorithms using case counts of 41 training provinces over 10 years (120 months) as the observed response matrix, leading to a training set of 4,920 instances (41 provinces × 120 months). We then tested the trained models by predicting observed case counts of 40 test provinces for the last two years (24 months), leading to a test set of 960 instances (40 provinces × 24 months).

The observed case counts were mapped to logarithmic scale after adding one since they are count data and contain zero values. These mapped values were used as the response matrix for all three algorithms. After training the algorithms, their predictions were mapped back to the original scale by exponentiating first and then subtracting one.

For RFR algorithm, we used the randomForest R package version 4.6-12 [38]. We set the formula parameter formula to “cases ~ year + month + season + latitude + longitude” to describe the model and set the number of trees to grow parameter ntree to 100,000, and other parameters were held at their default values.

For BRT algorithm, we used the gbm R package version 2.1.1 [39]. We set the formula parameter formula to “cases ~ year + month + season + latitude + longitude” to describe the model, set the maximum number of iterations (i.e., the maximum number of trees) parameter n.trees to 100,000, set the number of cross-validation folds parameter cv.folds to 5 and set the maximum depth of variable interactions parameter interaction.depth to 2, and other parameters were held at their default values.

We implemented our structured GPR algorithm in R and used the Gaussian kernel to define similarity functions on spatial and temporal covariates. The Gaussian kernel function $k_{G}(\cdot ,\cdot )$ between two data instances ***x***_*i*_ and ***x***_*j*_ can be defined as

$$\begin{array}{c} k_{G}\left(x_{i},x_{j}\right)=\exp(-∥x_{i}-x_{j}{∥}_{2}^{2}/s^{2}), \end{array}$$

where ‖ ⋅ ‖_2_ denotes the *ℓ*_2_ norm, and *s* is the kernel width parameter. For spatial covariates of two data instances (i.e., latitude and longitude coordinates of two province centres), we defined the spatial kernel as $k_{s}(s_{l},s_{m})=k_{G}(s_{l},s_{m})$ and picked the kernel width parameter as the mean of pairwise Euclidean distances between training instances. For temporal covariates of two time periods (i.e., years, months, and seasonal groups of two time periods), we defined the temporal kernel as the multiplication of three kernels, i.e., *k*_*t*_(***t***_*p*_, ***t***_*q*_) = *k*_year_(***t***_*p*_, ***t***_*q*_) *k*_month_(***t***_*p*_, ***t***_*q*_) k_season_(***t***_*p*_, ***t***_*q*_), to capture the interaction effects between them, where we had three separate Gaussian kernels on year, month, and seasonal group covariates. The kernel width parameters were chosen as the means of pairwise Euclidean distances between training instances for all three kernels. We picked the standard deviation parameter of measurement noise values *σ*_*y*_ as the standard deviation of log-scaled observed case counts of training instances.

We used the Pearson’s correlation coefficient (PCC) and normalized root mean squared error (NRMSE) to compare prediction performances of the three algorithms. PCC can be calculated as

$$\begin{array}{c} \text{PCC}=\frac{{\left(y-1y_{.}\right)}^{⊤}\left(\hat{y}-1{\hat{y}}_{.}\right)}{\sqrt{{\left(y-1y_{.}\right)}^{⊤}\left(y-1y_{.}\right)}\sqrt{{\left(\hat{y}-1{\hat{y}}_{.}\right)}^{⊤}\left(\hat{y}-1{\hat{y}}_{.}\right)}} \end{array}$$

where ***y*** and $\hat{y}$ denote the vectors of observed and predicted case counts, respectively, and *y*. and ${\hat{y}}_{.}$ denote the averages of ***y*** and $\hat{y}$, respectively. Larger PCC values correspond to better performance in capturing the trend in case counts. NRMSE can be calculated as

$$\begin{array}{c} \text{NRMSE}=\sqrt{\frac{{\left(y-\hat{y}\right)}^{⊤}\left(y-\hat{y}\right)}{{\left(y-1y_{.}\right)}^{⊤}\left(y-1y_{.}\right)}}. \end{array}$$

Smaller NRMSE values correspond to better performance in capturing the scale of case counts.

## Results

### Performance comparison

Table 1 reports PCC values of RFR, BRT, and GPR algorithms on our CCHF data set for three prediction scenarios. We see that GPR algorithm obtained the best PCC values by improving the results of temporal, spatial, and spatiotemporal prediction scenarios by 1.05%, 26.31%, and 16.45%, respectively. Note that RFR and BRT algorithms failed to capture the spatial spread of CCHF when predicting case counts for unseen provinces (i.e., in spatial and spatiotemporal scenarios), whereas GPR algorithm was able to capture this spread by obtaining more than 70% PCC for these two scenarios. All algorithms achieved PCC values around 75% and 85% for temporal scenario since capturing temporal dynamics is easier owing to annual periodicity of CCHF cases.

**Table 1:** Pearson’s correlation coefficients of three algorithms on CCHF data set for three prediction scenarios together with ranks in parentheses.

	Temporal	Spatial	Spatiotemporal
**RFR**	0.748 (3)	0.486 (2)	0.543 (2)
**BRT**	0.846 (2)	0.437 (3)	0.493 (3)
**GPR**	0.857 (1)	0.749 (1)	0.707 (1)

Table 2 shows NRMSE values of RFR, BRT, and GPR algorithms on our CCHF data set for temporal, spatial, and spatiotemporal prediction scenarios. We see that GPR algorithm again obtained the best NRMSE values by improving the results of temporal, spatial, and spatiotemporal prediction scenarios by 21.39%, 20.38% and 15.65%, respectively. Even though BRT algorithm obtained a PCC value comparable to that of GPR algorithm for temporal scenario, GPR algorithm obtained considerably better NRMSE values than both RFR and BRT algorithms. This shows that GPR algorithm is better than the other two algorithms in terms of capturing the range of CCHF cases in the test sets as discussed below.

**Table 2:** Normalized root mean squared errors of three algorithms on CCHF data set for three prediction scenarios together with ranks in parentheses.

	Temporal	Spatial	Spatiotemporal
**RFR**	0.875 (3)	0.927 (3)	0.894 (3)
**BRT**	0.746 (2)	0.900 (2)	0.876 (2)
**GPR**	0.532 (1)	0.697 (1)	0.720 (1)

**Fig 5:**
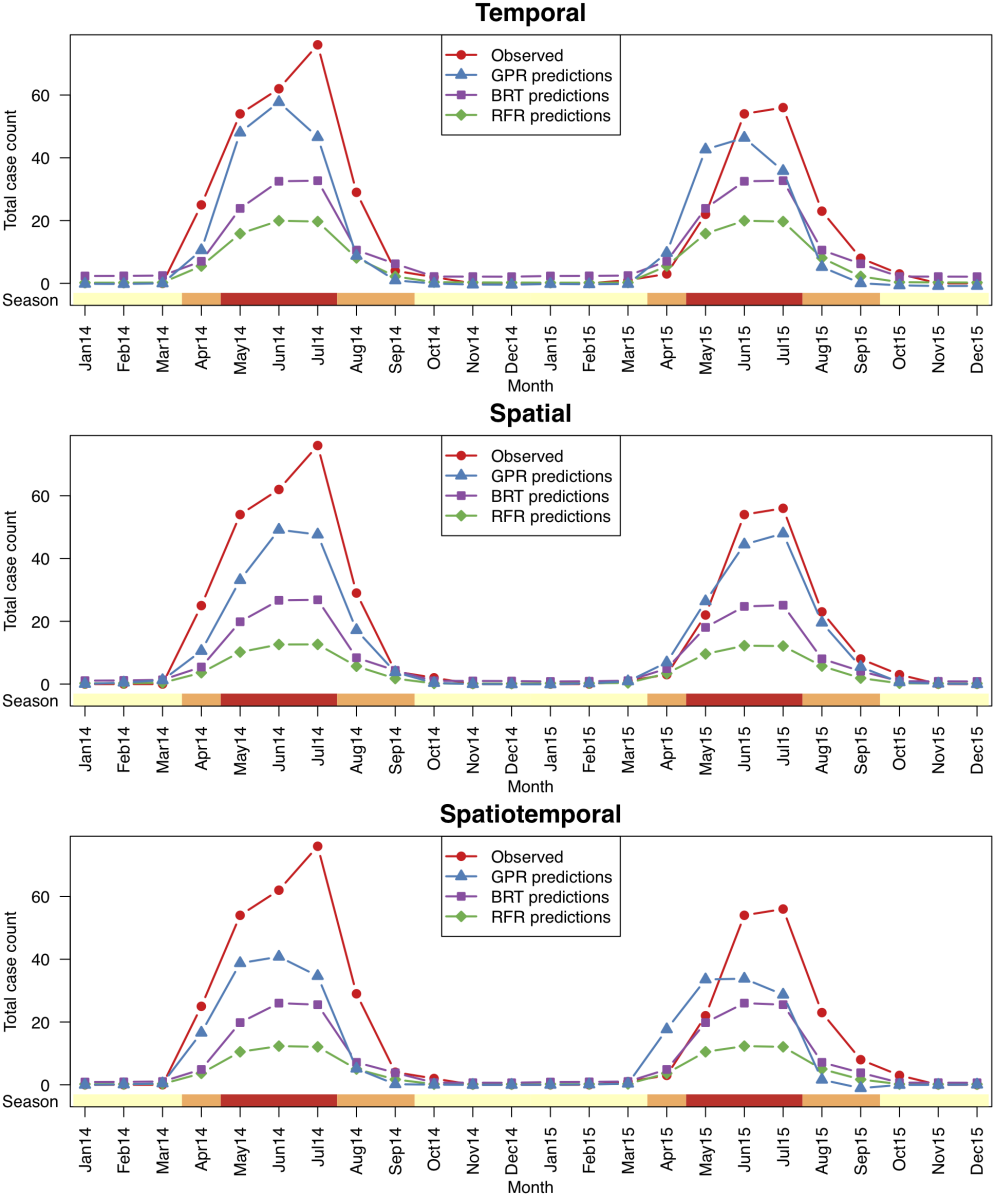
The total observed and predicted case counts by each algorithm for years 2014 and 2015 over the five provinces with the highest case counts (i.e., endemic region) among 40 common test provinces of all scenarios. The time periods were annotated by their seasonal group information at the bottom (yellow: cold; orange: warm; red: hot). Note that all three algorithms were able to capture the annual periodicity of CCHF cases in all scenarios, whereas the predicted case counts of GPR algorithm were closer to the observed CCHF cases.

Fig 5 shows the total observed and predicted case counts by RFR, BRT and GPR algorithms for years 2014 and 2015 over the five provinces with the highest case counts among 40 common test provinces of all scenarios. We see that all three algorithms captured the annual periodicity of CCHF cases, whereas GPR algorithm performed the best in terms of predicting the observed case counts. RFR algorithm was not able to predict the observed case counts owing to its lack of high order interactions between covariates, whereas BRT algorithm performed better owing to its second order interactions. The same results were also valid if we took the first 10, 15, and 20 provinces from 40 common test provinces (S14, S15 and S16 Figs).

S17 Fig gives a detailed comparison between observed and predicted case counts of RFR, BRT, and GPR algorithms for the same five provinces reported in Fig 5. We see that GPR algorithm produced predictions mostly in agreement with the range of observed CCHF case counts, whereas RFR and BRT algorithms underestimated CCHF case counts in most of the time periods. BRT algorithm obtained NRMSE value comparable to that of GPR algorithm for temporal scenario, whereas GPR algorithm reduced NRMSE values by 0.277 and 0.170 for spatial and spatiotemporal scenarios, respectively.

The results of the computational experiments reported in this study can be analyzed from different perspectives. We analyzed the results with respect to prediction scenarios, machine learning algorithms, computational complexity, dependency on training set size, and dependency on sampling over provinces.

### Prediction scenarios

We performed computational experiments under three different scenarios. As we can see from Tables 1, 2, Fig 5 and S17 Fig, making temporal predictions (i.e., predicting future time periods by looking at the historical data) is strikingly easier than making spatial and spatiotemporal predictions (i.e., generalizing to unseen locations). Most infectious disease outbreaks occur in cycles (i.e., ascending, plateau, and descending phases), and this structure makes temporal prediction easier. The disease we addressed is a vector-borne infectious disease mainly transmitted by infected tick bites, leading to a strong temporal dependency owing to the sleep cycles of ticks.

### Machine learning algorithms

We used three machine learning algorithms for predicting case counts. As we discussed before, GPR algorithm was able to capture the range of CCHF case counts better than RFR and BRT algorithms. We think that this was mainly due to the capability of GPR algorithm to model highly complex dependencies between input and output covariates thanks to nonlinear kernel functions such as the Gaussian kernel we used. We also noted from Fig 5 and S17 Fig that the main improvement of GPR algorithm over the others was the ability to better capture the range of case counts in the time periods with nonzero observed case counts. In the literature, RFR and BRT algorithms were frequently used as classification algorithms to predict whether there will be cases. In terms of classification performance, we would not expect major differences between three algorithms.

### Computational complexity

Instead of using a naive version of GPR algorithm, we implemented an efficient variant that exploits the special structure of the kernel matrix to make inference very fast. We decomposed the kernel matrix into a Kronecker product of two smaller kernel matrices calculated on spatial and temporal covariates, respectively. By doing so, we were able to perform inference for our structured GPR formulation in the order of milliseconds, whereas RFR and BRT algorithms took several minutes to complete using drastically higher physical memory.

### Dependency on training set size

To show the dependency of GPR on training set size, we performed an additional set of experiments by changing the number of years used for training. We used CCHF case counts of the last two, four, six, eight, and ten years between 2004 and 2013, respectively. Table 3 shows PCC and NRMSE values of GPR algorithm for this new set of experiments. We can see that there was an increasing trend in predictive performance as we increased the training set size.

**Table 3:** Pearson’s correlation coefficients and normalized root mean squared errors of GPR algorithm on CCHF data set with changing training set size (i.e., 2, 4, 6, 8, and 10 years).

	Temporal		Spatiotemporal	
	PCC	NRMSE	PCC	NRMSE
2012–13	0.633	1.015	0.558	1.039
2010–13	0.749	0.830	0.636	0.960
2008–13	0.831	0.582	0.725	0.760
2006–13	0.791	0.637	0.745	0.671
2004–13	0.857	0.532	0.707	0.720

### Dependency on sampling over provinces

**Fig 6:**
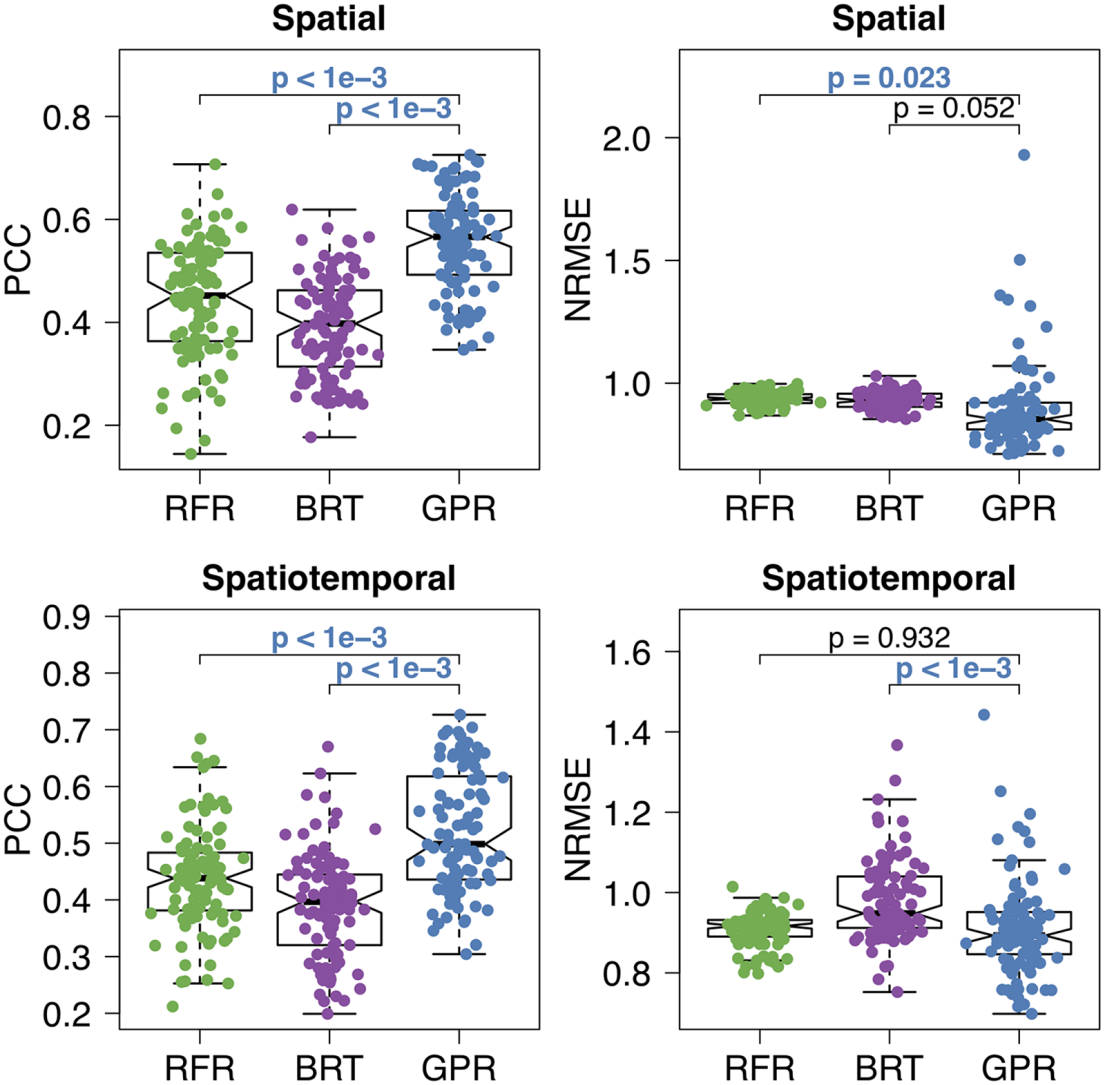
Pearson’s correlation coefficients and normalized root mean squared errors of three algorithms on CCHF data set for 100 different training and test set splits of 81 provinces for spatial and spatiotemporal modeling scenarios. GPR was compared against RFR and BRT using a two-sided paired *t*-test to check whether the predictive performances are significantly different, and *p*-value for each comparison was also reported. If the *p*-value is less than 0.05, it is typeset with the color of the winning algorithm.

Up to this point, we performed our experiments on a fixed training and test set split (S13 Fig), which was designed to make training and test sets as similar as possible, to better illustrate the differences between machine learning algorithms. We also compared the predictive performances of RFR, BRT, and GPR on 100 different training and set set splits constructed by random sampling on 81 provinces. Fig 6 shows PCC and NRMSE values of the algorithms for spatial and spatiotemporal modeling scenarios. We see that our algorithm GPR was statistically significantly better (i.e., *p* < 0.001) than other two algorithms for both scenarios in terms of PCC values. In spatial prediction scenario, GPR achieved statistically significantly better NRMSE values than RFR (i.e., *p* = 0.023), but it obtained NRMSE values comparable to BRT (i.e., *p* = 0.052). In spatiotemporal prediction scenario, NRMSE values of GPR were statistically significantly better than those of BRT (i.e., *p* < 0.001), whereas NRMSE values were comparable between GPR and RFR (i.e., *p* = 0.932).

## Discussion

Infectious diseases cause important health problems worldwide and create difficult challenges for public health policy makers. To be able to make correct and effective decisions, it is quite important to understand the characteristics of each infectious disease, which includes environmental factors such as climate and animal population in addition to molecular evolution of disease sources such as bacteria and viruses. In this study, we addressed to capture the effect of environmental factors on infectious diseases by modeling their spatial and temporal dependencies on these factors.

For this purpose, several computational methods have been proposed in the literature, whereas we focused only on machine learning algorithms applied to this problem. Easy-to-use machine learning algorithms such as random forests and boosted regression trees were frequently used in infectious disease modeling studies. However, Gaussian processes might capture highly complex dependencies better than these tree-based algorithms. Thus, we formulated a computational framework based on Gaussian processes that can be used to perform spatial, temporal, or spatiotemporal prediction of infectious diseases.

We integrated spatial features (such as geographical coordinates) and temporal features (such as seasonal conditions) for location and time period pairs that were used as data instances in our Gaussian process formulation. However, a naive implementation of Gaussian processes would become computationally infeasible owing to very high numbers of pairs being modeled. We exploited the special structure (i.e., Kronecker) of similarity matrices in our formulation to obtain a very efficient implementation, which enabled us to train models for around 10,000 data instances in the order of milliseconds.

We applied our framework to the problem of predicting the case counts of a vector-borne infectious disease Crimean–Congo hemorrhagic fever using the data set of infected case counts between years 2004 and 2015 collected by the Ministry of Health of Turkey. We performed predictions under three different scenarios (Fig 1), which correspond to making predictions for unseen provinces (i.e., spatial prediction), future time periods (i.e., temporal prediction), or unseen province and time period pairs (i.e., spatiotemporal prediction) to show the suitability of our approach to distinct problems.

Predicting future cases of infectious diseases is very important for the control and prevention of the disease. The predicted case counts can be used to develop new public health policies and intervention mechanisms. It is more useful for public health policy makers to be able to predict the possible number of infected cases for a region and a time period pair rather than predicting whether there will be cases or not. Policy makers can make use of predicted number of infected cases to purchase vaccines around the right amount, to raise public awareness in the region, to educate healthcare workers, etc. From that perspective, GPR algorithm did a better job than RFR and BRT algorithms by predicting CCHF case counts more accurately (i.e., lower NRMSE values).

We tested our proposed formulation on a single disease, but the same framework can be extended towards other vector-borne infectious diseases (e.g., dengue fever, malaria, Zika fever) and as well as other infectious diseases (e.g., influenza, measles, tuberculosis). We also made the source code publicly available to enable other computational and applied researchers to make such extensions easily.

## Supporting information

S1 Fig The total numbers of infected cases reported in 81 provinces of Turkey during 2004. The numbers were shown on the province centers. This map was generated using the Turkish administrative map downloaded from https://www.gadm.org and the R package maps version 3.3.0 at https://cran.r-project.org/web/packages/maps.
(TIFF)

S2 Fig The total numbers of infected cases reported in 81 provinces of Turkey during 2005. The numbers were shown on the province centers. This map was generated using the Turkish administrative map downloaded from https://www.gadm.org and the R package maps version 3.3.0 at https://cran.r-project.org/web/packages/maps.
(TIFF)

S3 Fig The total numbers of infected cases reported in 81 provinces of Turkey during 2006. The numbers were shown on the province centers. This map was generated using the Turkish administrative map downloaded from https://www.gadm.org and the R package maps version 3.3.0 at https://cran.r-project.org/web/packages/maps.
(TIFF)

S4 Fig The total numbers of infected cases reported in 81 provinces of Turkey during 2007. The numbers were shown on the province centers. This map was generated using the Turkish administrative map downloaded from https://www.gadm.org and the R package maps version 3.3.0 at https://cran.r-project.org/web/packages/maps.
(TIFF)

S5 Fig The total numbers of infected cases reported in 81 provinces of Turkey during 2008. The numbers were shown on the province centers. This map was generated using the Turkish administrative map downloaded from https://www.gadm.org and the R package maps version 3.3.0 at https://cran.r-project.org/web/packages/maps.
(TIFF)

S6 Fig The total numbers of infected cases reported in 81 provinces of Turkey during 2009. The numbers were shown on the province centers. This map was generated using the Turkish administrative map downloaded from https://www.gadm.org and the R package maps version 3.3.0 at https://cran.r-project.org/web/packages/maps.
(TIFF)

S7 Fig The total numbers of infected cases reported in 81 provinces of Turkey during 2010. The numbers were shown on the province centers. This map was generated using the Turkish administrative map downloaded from https://www.gadm.org and the R package maps version 3.3.0 at https://cran.r-project.org/web/packages/maps.
(TIFF)

S8 Fig The total numbers of infected cases reported in 81 provinces of Turkey during 2011. The numbers were shown on the province centers. This map was generated using the Turkish administrative map downloaded from https://www.gadm.org and the R package maps version 3.3.0 at https://cran.r-project.org/web/packages/maps.
(TIFF)

S9 Fig The total numbers of infected cases reported in 81 provinces of Turkey during 2012. The numbers were shown on the province centers. This map was generated using the Turkish administrative map downloaded from https://www.gadm.org and the R package maps version 3.3.0 at https://cran.r-project.org/web/packages/maps.
(TIFF)

S10 Fig The total numbers of infected cases reported in 81 provinces of Turkey during 2013. The numbers were shown on the province centers. This map was generated using the Turkish administrative map downloaded from https://www.gadm.org and the R package maps version 3.3.0 at https://cran.r-project.org/web/packages/maps.
(TIFF)

S11 Fig The total numbers of infected cases reported in 81 provinces of Turkey during 2014. The numbers were shown on the province centers. This map was generated using the Turkish administrative map downloaded from https://www.gadm.org and the R package maps version 3.3.0 at https://cran.r-project.org/web/packages/maps.
(TIFF)

S12 Fig The total numbers of infected cases reported in 81 provinces of Turkey during 2015. The numbers were shown on the province centers. This map was generated using the Turkish administrative map downloaded from https://www.gadm.org and the R package maps version 3.3.0 at https://cran.r-project.org/web/packages/maps.
(TIFF)

S13 Fig Training and test set split of 81 provinces for spatial and spatiotemporal modeling scenarios. Red-colored 41 provinces were used as the training set, whereas gray-colored 40 provinces were used as the test test. Province IDs were shown on the province centers. This map was generated using the Turkish administrative map downloaded from https://www.gadm.org and the R package maps version 3.3.0 at https://cran.r-project.org/web/packages/maps.
(TIFF)

S14 Fig The total observed and predicted case counts by each algorithm for years 2014 and 2015 over the 10 provinces with the highest case counts among 40 common test provinces of all scenarios. The time periods were annotated by their seasonal group information at the top (yellow: cold; orange: warm; red: hot).
(TIFF)

S15 Fig The total observed and predicted case counts by each algorithm for years 2014 and 2015 over the 15 provinces with the highest case counts among 40 common test provinces of all scenarios. The time periods were annotated by their seasonal group information at the top (yellow: cold; orange: warm; red: hot).
(TIFF)

S16 Fig The total observed and predicted case counts by each algorithm for years 2014 and 2015 over the 20 provinces with the highest case counts among 40 common test provinces of all scenarios. The time periods were annotated by their seasonal group information at the top (yellow: cold; orange: warm; red: hot).
(TIFF)

S17 Fig The observed (x-axis) and predicted case counts (y-axis) by each algorithm in time periods of years 2014 and 2015 for the five provinces with the highest case counts among 40 common test provinces of all scenarios. Each province was represented with a distinct marker. We also reported NRMSE values for each algorithm and scenario pair at the bottom-right corner. We also drew a dashed unit slope line to show whether the algorithms captured the range of observed CCHF case counts. Note that BRT and GPR algorithms obtained comparable results for temporal scenario, whereas GPR algorithm achieved remarkably better prediction performances than RFR and BRT algorithms under other two scenarios.
(TIFF)

S1 File Surveillance data set of 9,636 CCHF infection cases reported in Turkey between years 2004 and 2015, which was collected by the Ministry of Health of Turkey. Province IDs reported in this file correspond to numbers shown in S13 Fig.
(XLSX)
