# Supplementary figures and images for: Spatiotemporal prediction of infectious diseases using structured Gaussian processes with application to Crimean–Congo hemorrhagic fever
Source: PLoS Negl Trop Dis. 2018 Aug 17;12(8):e0006737. doi: 10.1371/journal.pntd.0006737 (PMC6114917; doi:10.1371/journal.pntd.0006737)

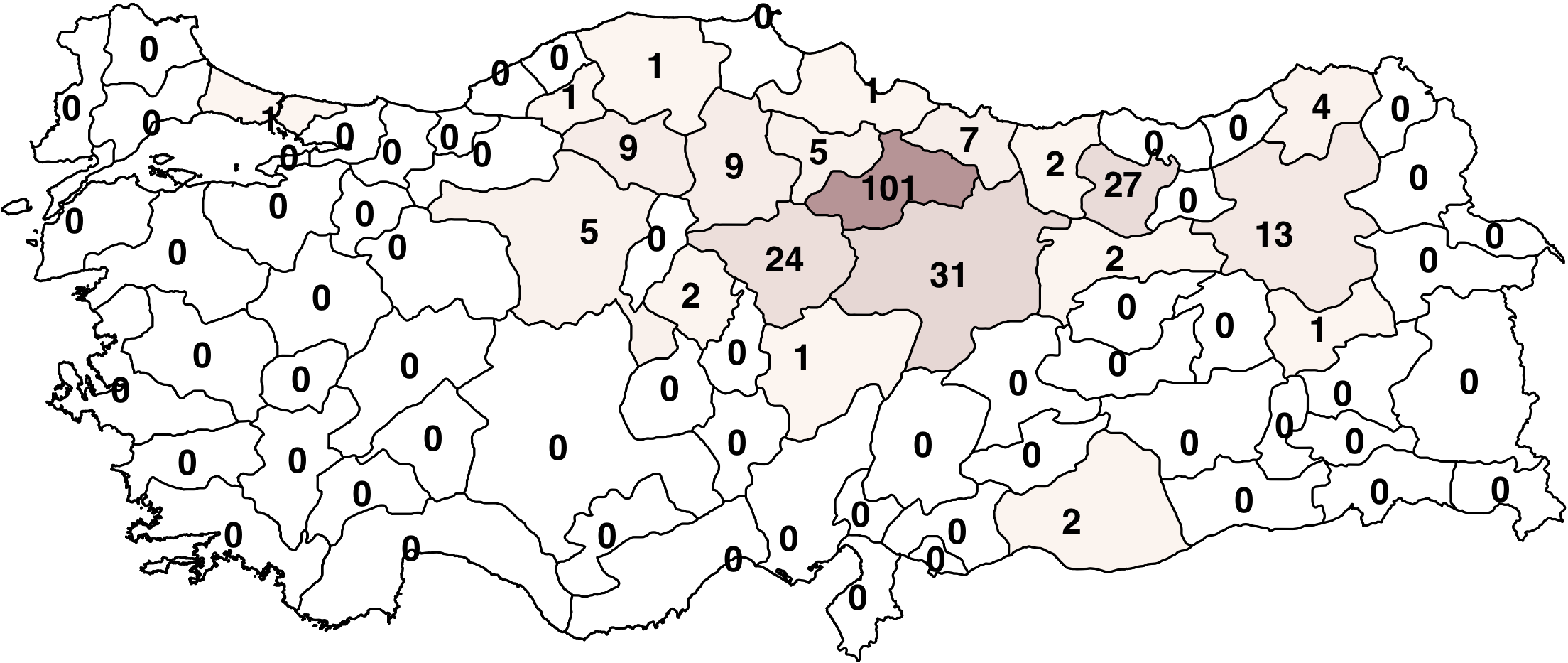

Supplement: S1 Fig — The numbers were shown on the province centers. This map was generated using the Turkish administrative map downloaded from https://www.gadm.org and the R package maps version 3.3.0 at https://cran.r-project.org/web/packages/maps. (TIFF) [file pntd.0006737.s001.tiff]

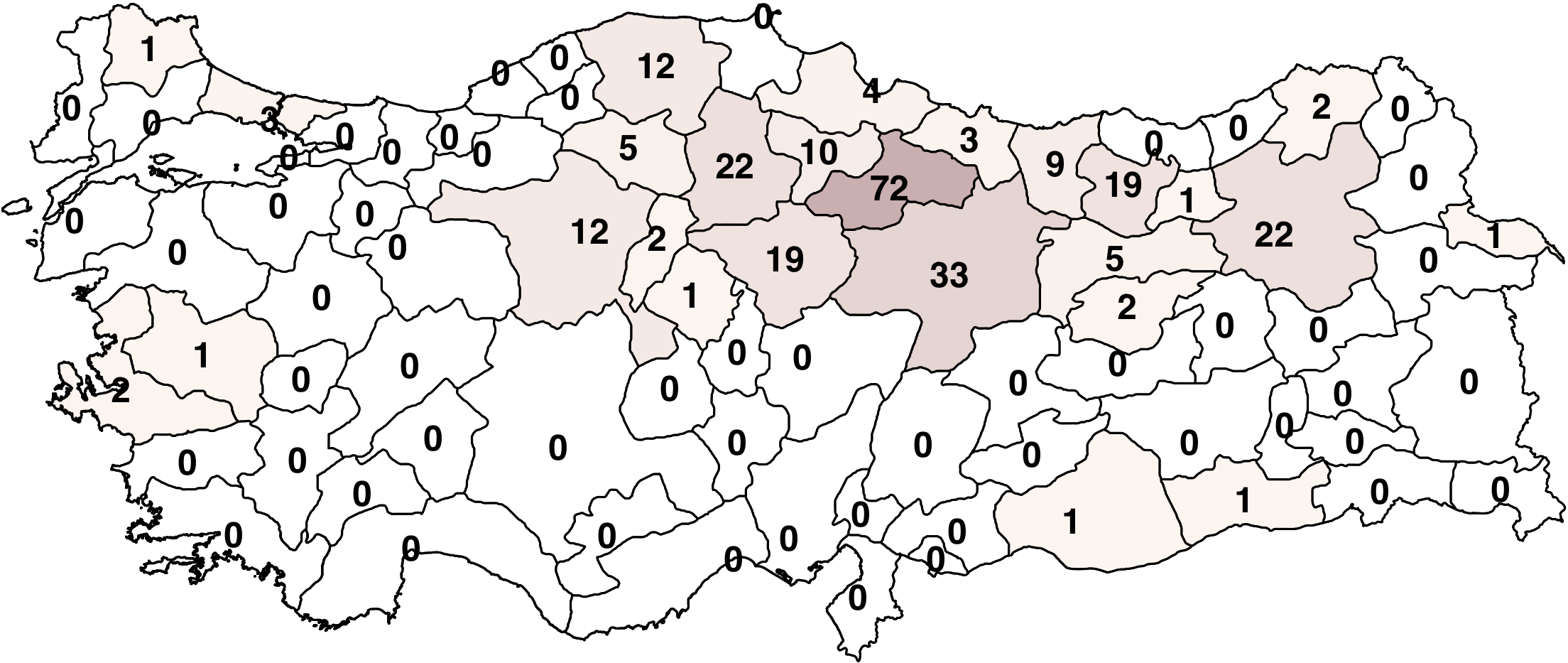

Supplement: S2 Fig — The numbers were shown on the province centers. This map was generated using the Turkish administrative map downloaded from https://www.gadm.org and the R package maps version 3.3.0 at https://cran.r-project.org/web/packages/maps. (TIFF) [file pntd.0006737.s002.tiff]

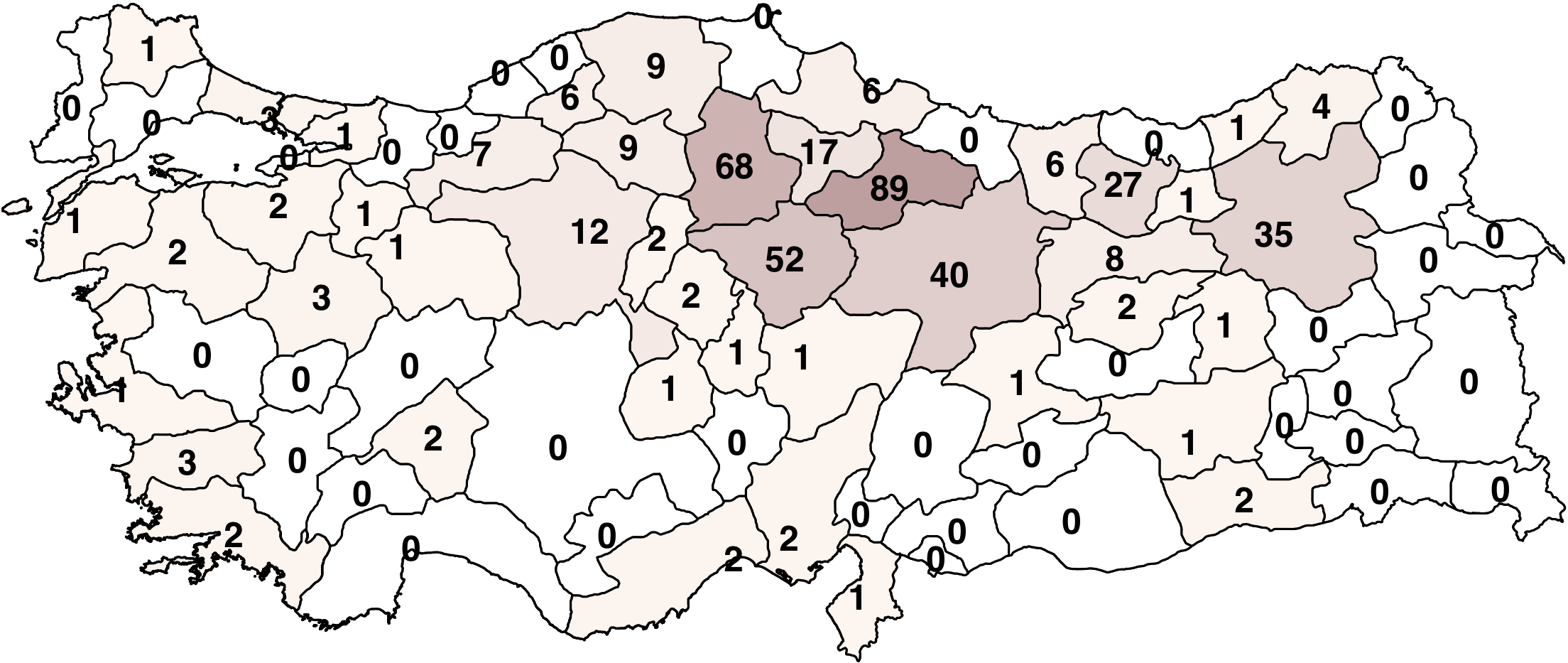

Supplement: S3 Fig — The numbers were shown on the province centers. This map was generated using the Turkish administrative map downloaded from https://www.gadm.org and the R package maps version 3.3.0 at https://cran.r-project.org/web/packages/maps. (TIFF) [file pntd.0006737.s003.tiff]

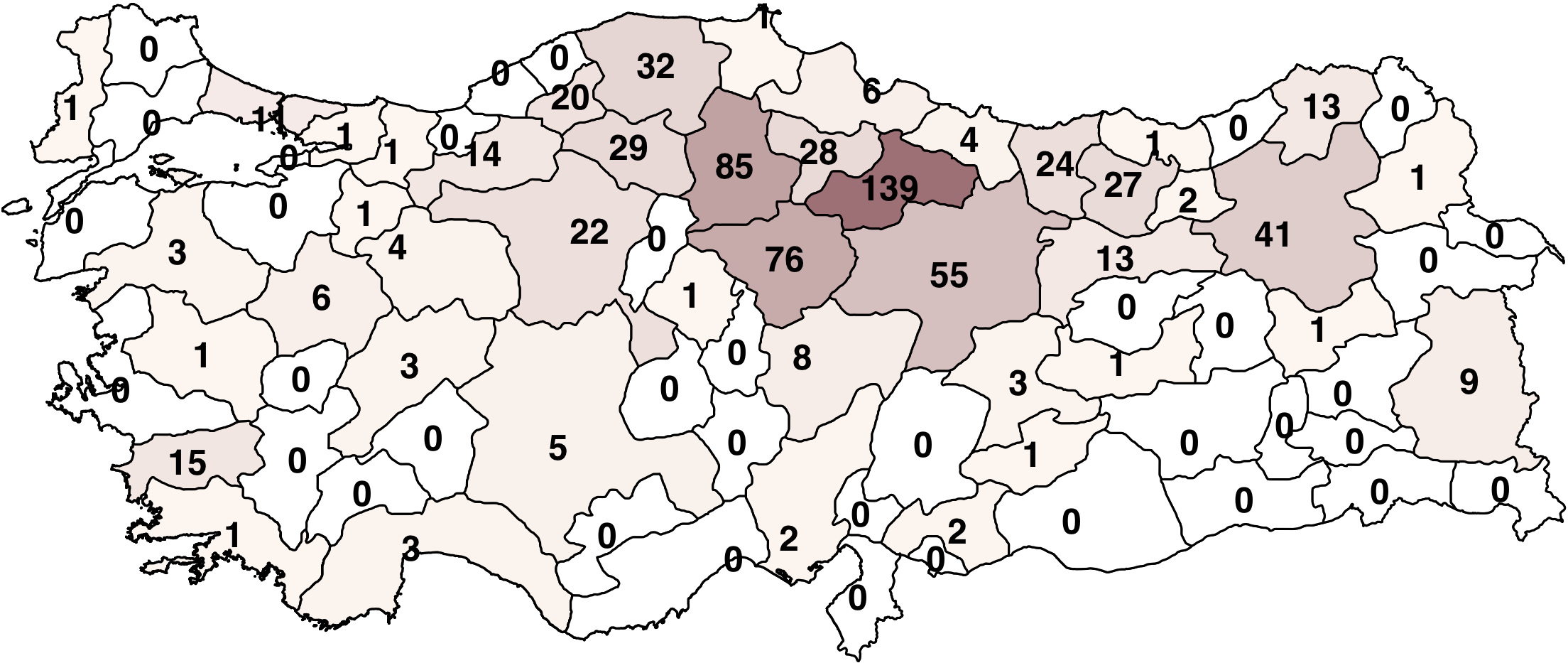

Supplement: S4 Fig — The numbers were shown on the province centers. This map was generated using the Turkish administrative map downloaded from https://www.gadm.org and the R package maps version 3.3.0 at https://cran.r-project.org/web/packages/maps. (TIFF) [file pntd.0006737.s004.tiff]

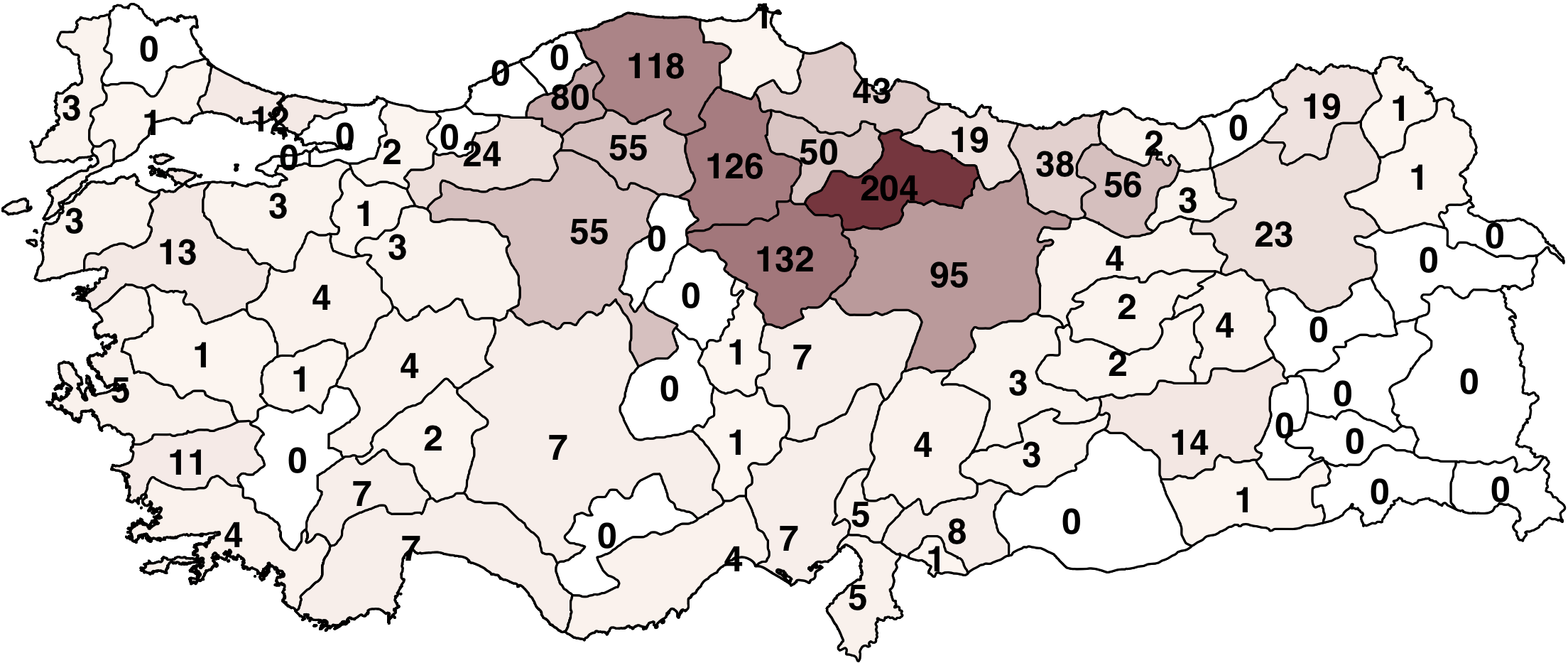

Supplement: S5 Fig — The numbers were shown on the province centers. This map was generated using the Turkish administrative map downloaded from https://www.gadm.org and the R package maps version 3.3.0 at https://cran.r-project.org/web/packages/maps. (TIFF) [file pntd.0006737.s005.tiff]

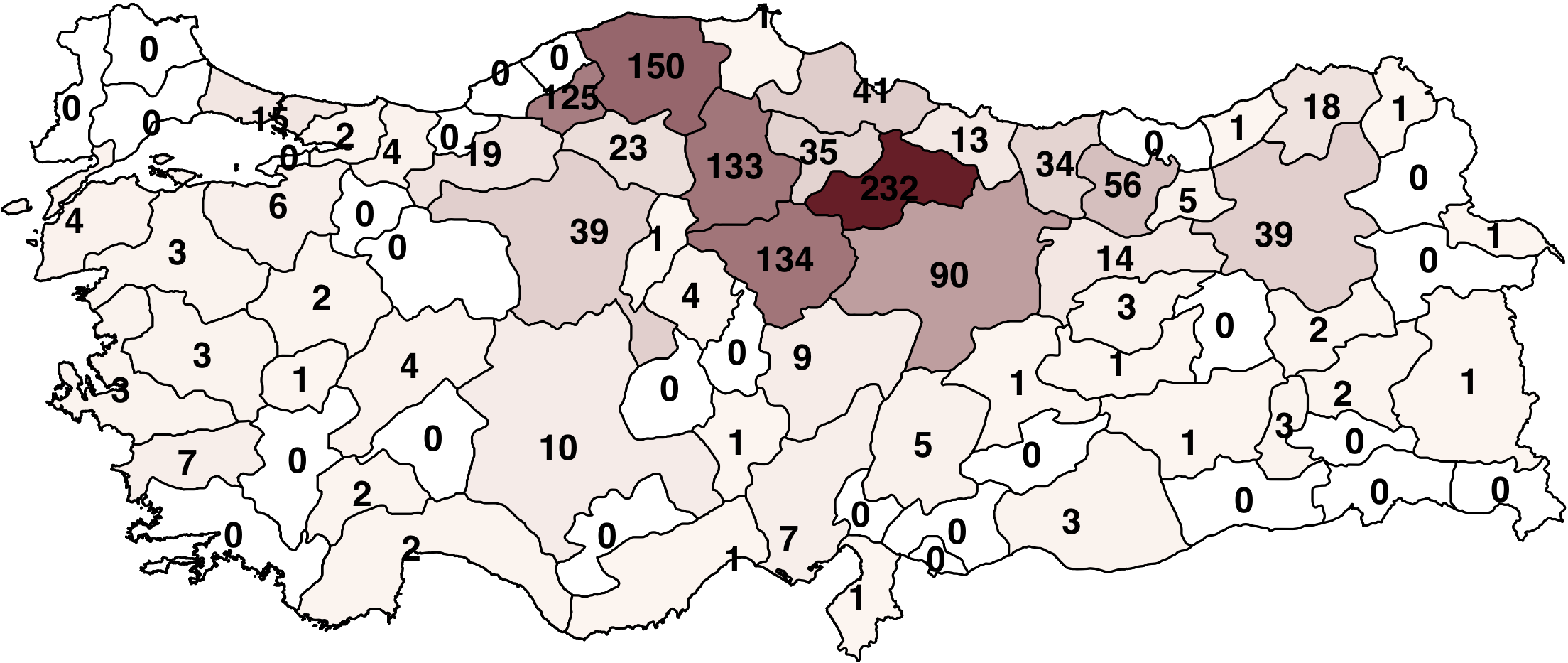

Supplement: S6 Fig — The numbers were shown on the province centers. This map was generated using the Turkish administrative map downloaded from https://www.gadm.org and the R package maps version 3.3.0 at https://cran.r-project.org/web/packages/maps. (TIFF) [file pntd.0006737.s006.tiff]

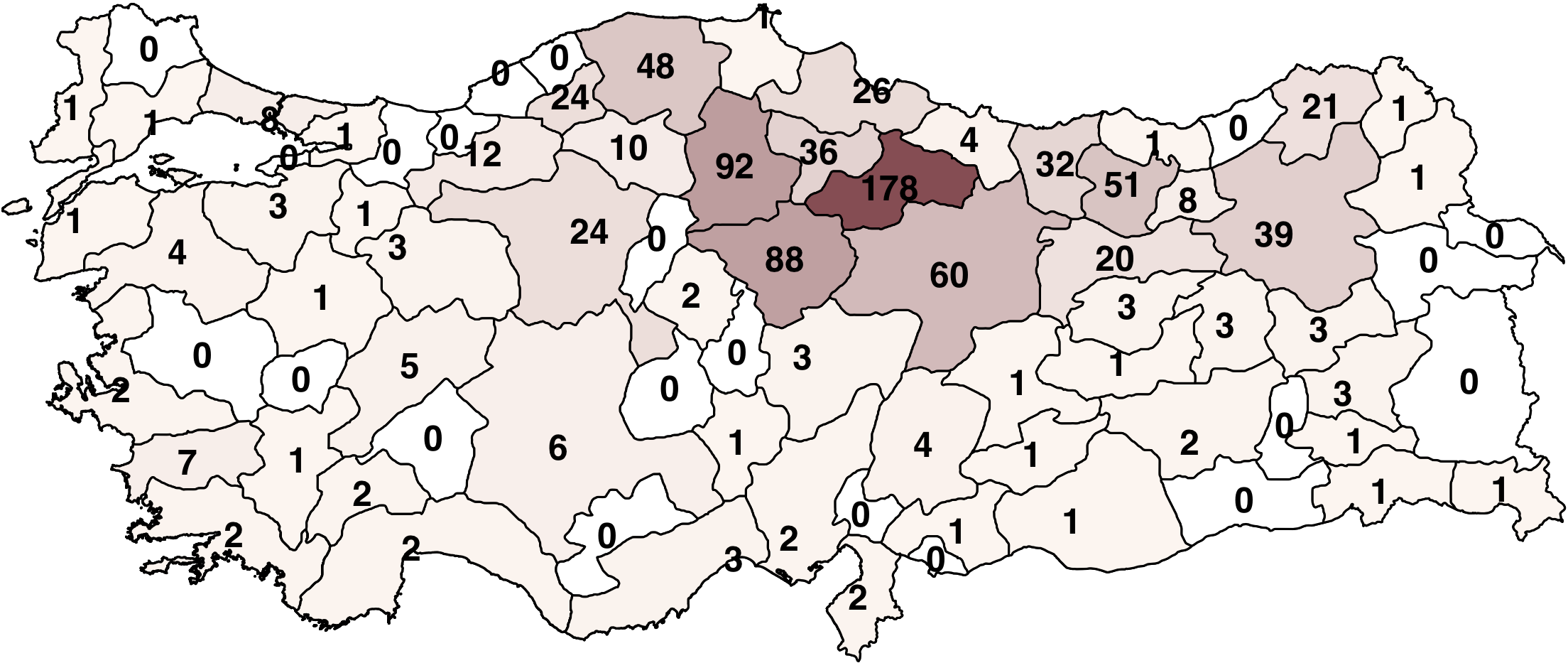

Supplement: S7 Fig — The numbers were shown on the province centers. This map was generated using the Turkish administrative map downloaded from https://www.gadm.org and the R package maps version 3.3.0 at https://cran.r-project.org/web/packages/maps. (TIFF) [file pntd.0006737.s007.tiff]

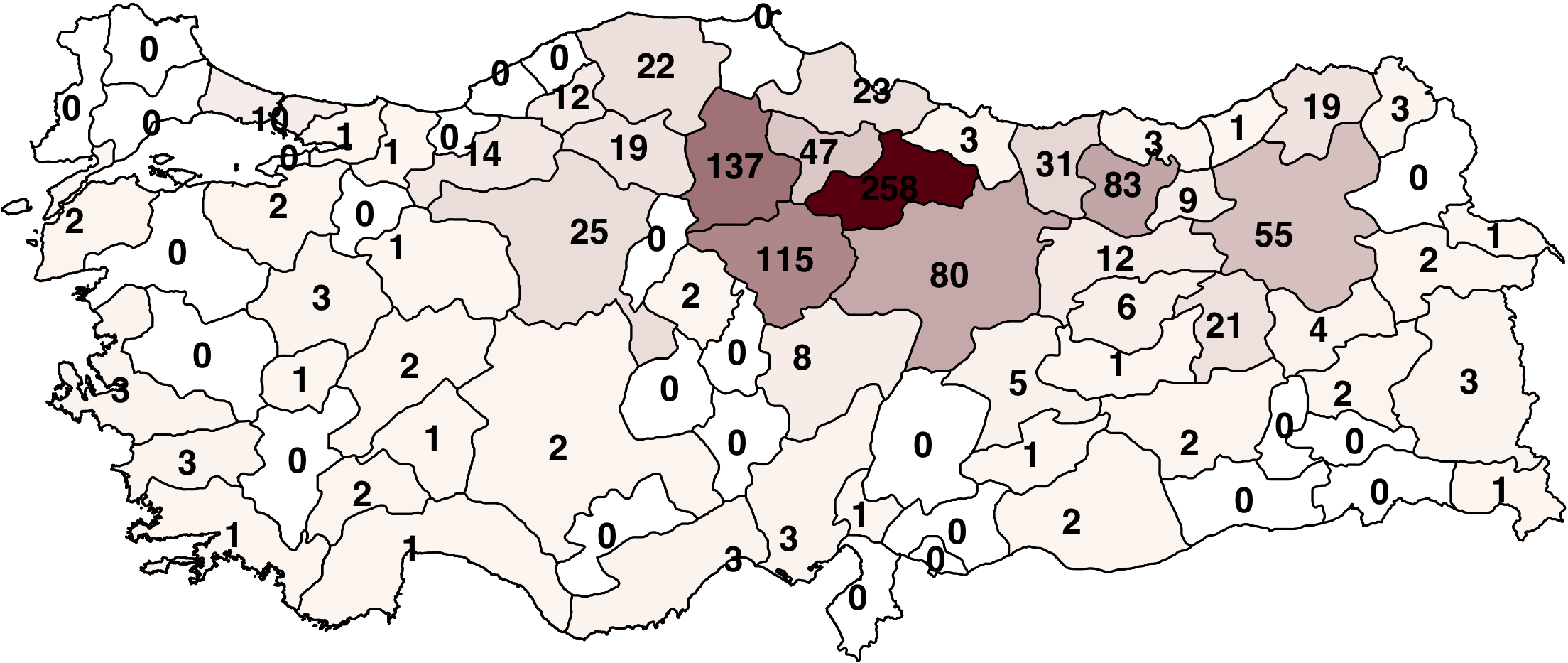

Supplement: S8 Fig — The numbers were shown on the province centers. This map was generated using the Turkish administrative map downloaded from https://www.gadm.org and the R package maps version 3.3.0 at https://cran.r-project.org/web/packages/maps. (TIFF) [file pntd.0006737.s008.tiff]

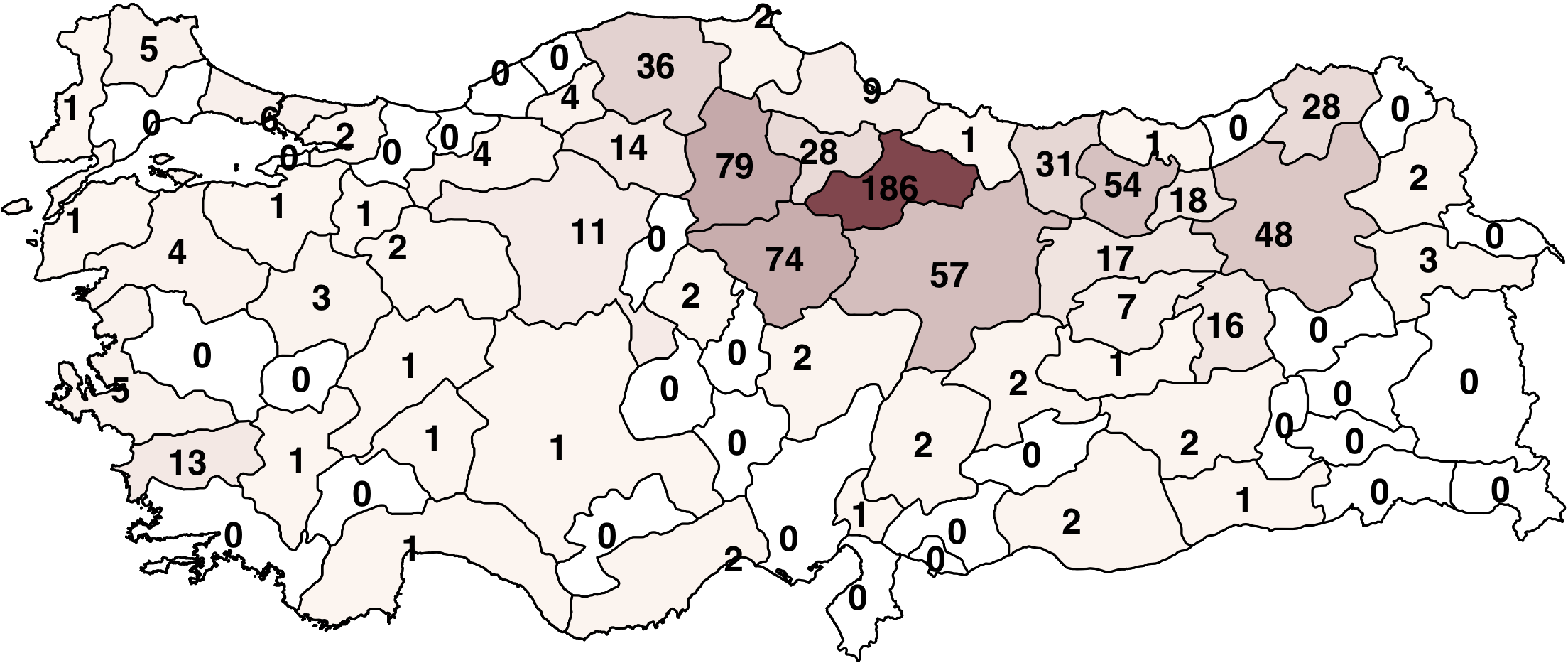

Supplement: S9 Fig — The numbers were shown on the province centers. This map was generated using the Turkish administrative map downloaded from https://www.gadm.org and the R package maps version 3.3.0 at https://cran.r-project.org/web/packages/maps. (TIFF) [file pntd.0006737.s009.tiff]

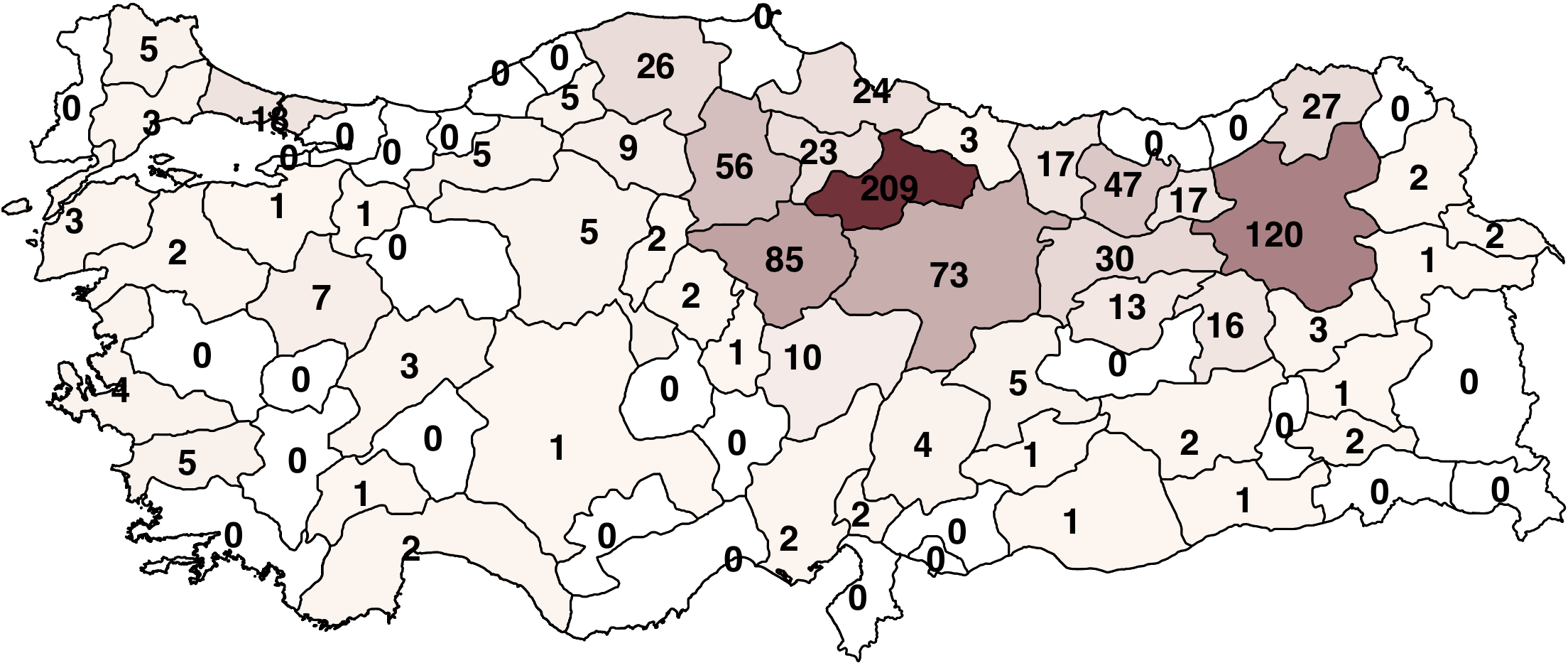

Supplement: S10 Fig — The numbers were shown on the province centers. This map was generated using the Turkish administrative map downloaded from https://www.gadm.org and the R package maps version 3.3.0 at https://cran.r-project.org/web/packages/maps. (TIFF) [file pntd.0006737.s010.tiff]

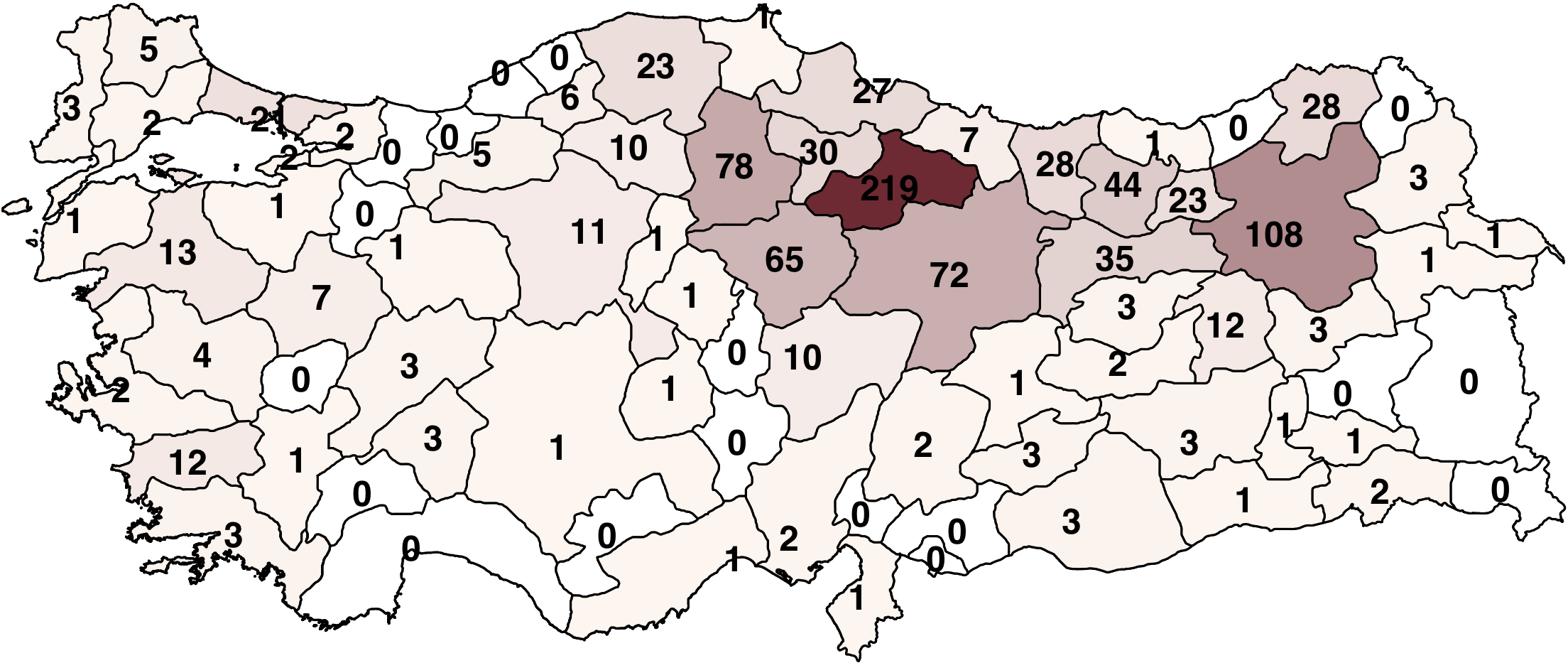

Supplement: S11 Fig — The numbers were shown on the province centers. This map was generated using the Turkish administrative map downloaded from https://www.gadm.org and the R package maps version 3.3.0 at https://cran.r-project.org/web/packages/maps. (TIFF) [file pntd.0006737.s011.tiff]

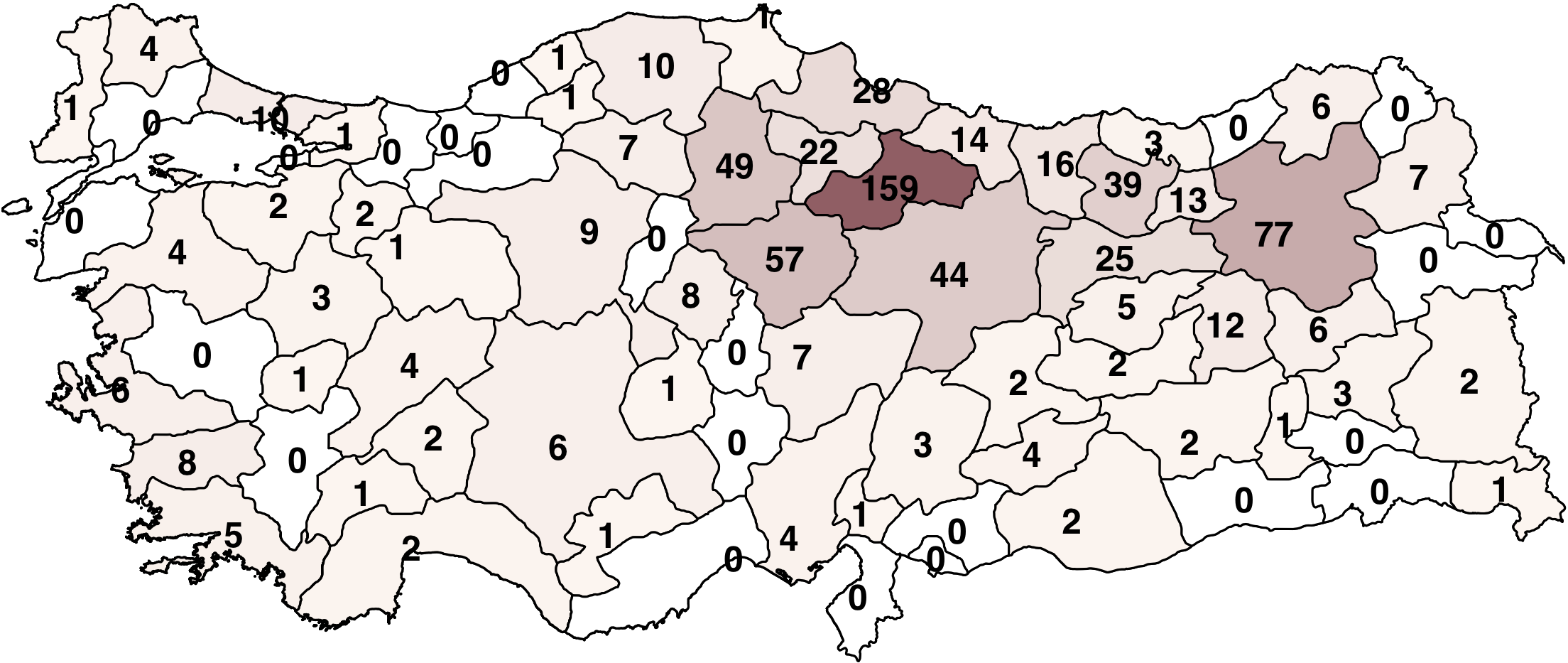

Supplement: S12 Fig — The numbers were shown on the province centers. This map was generated using the Turkish administrative map downloaded from https://www.gadm.org and the R package maps version 3.3.0 at https://cran.r-project.org/web/packages/maps. (TIFF) [file pntd.0006737.s012.tiff]

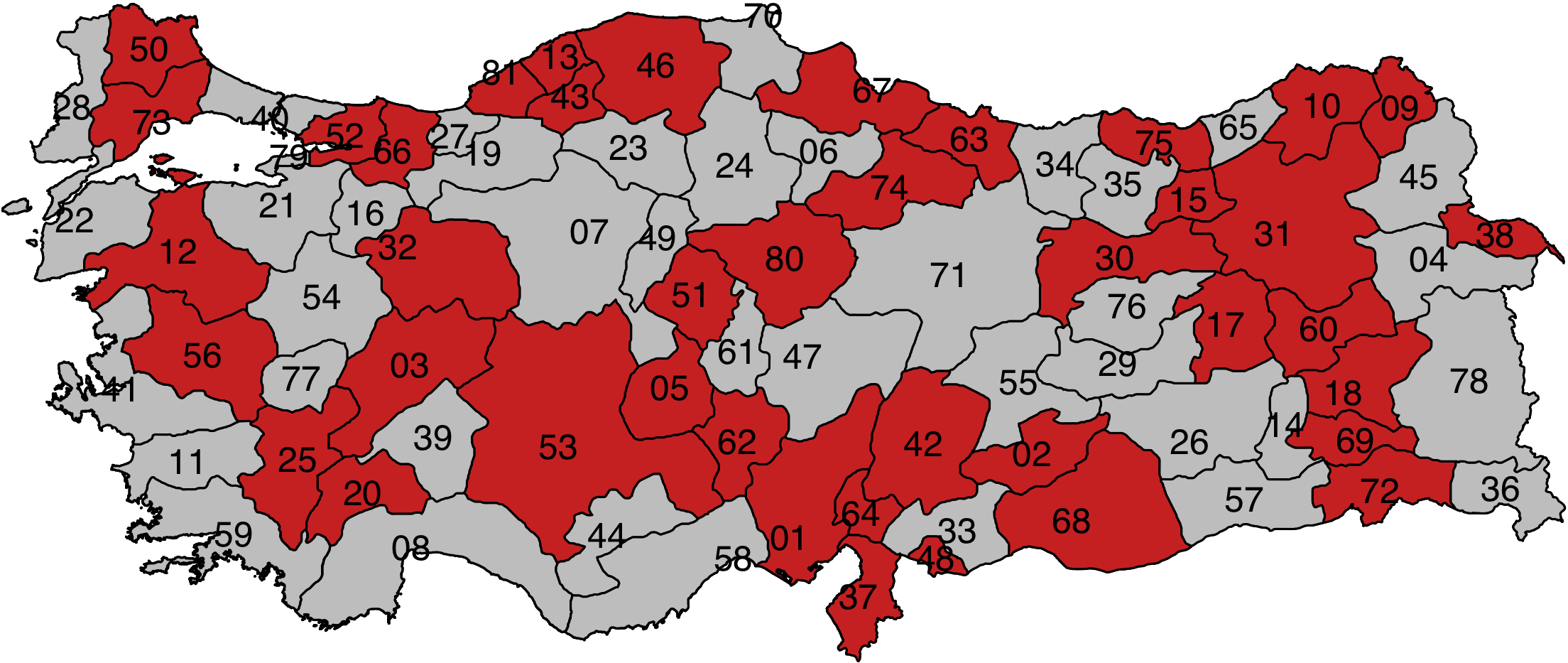

Supplement: S13 Fig — Red-colored 41 provinces were used as the training set, whereas gray-colored 40 provinces were used as the test test. Province IDs were shown on the province centers. This map was generated using the Turkish administrative map downloaded from https://www.gadm.org and the R package maps version 3.3.0 at https://cran.r-project.org/web/packages/maps. (TIFF) [file pntd.0006737.s013.tiff]

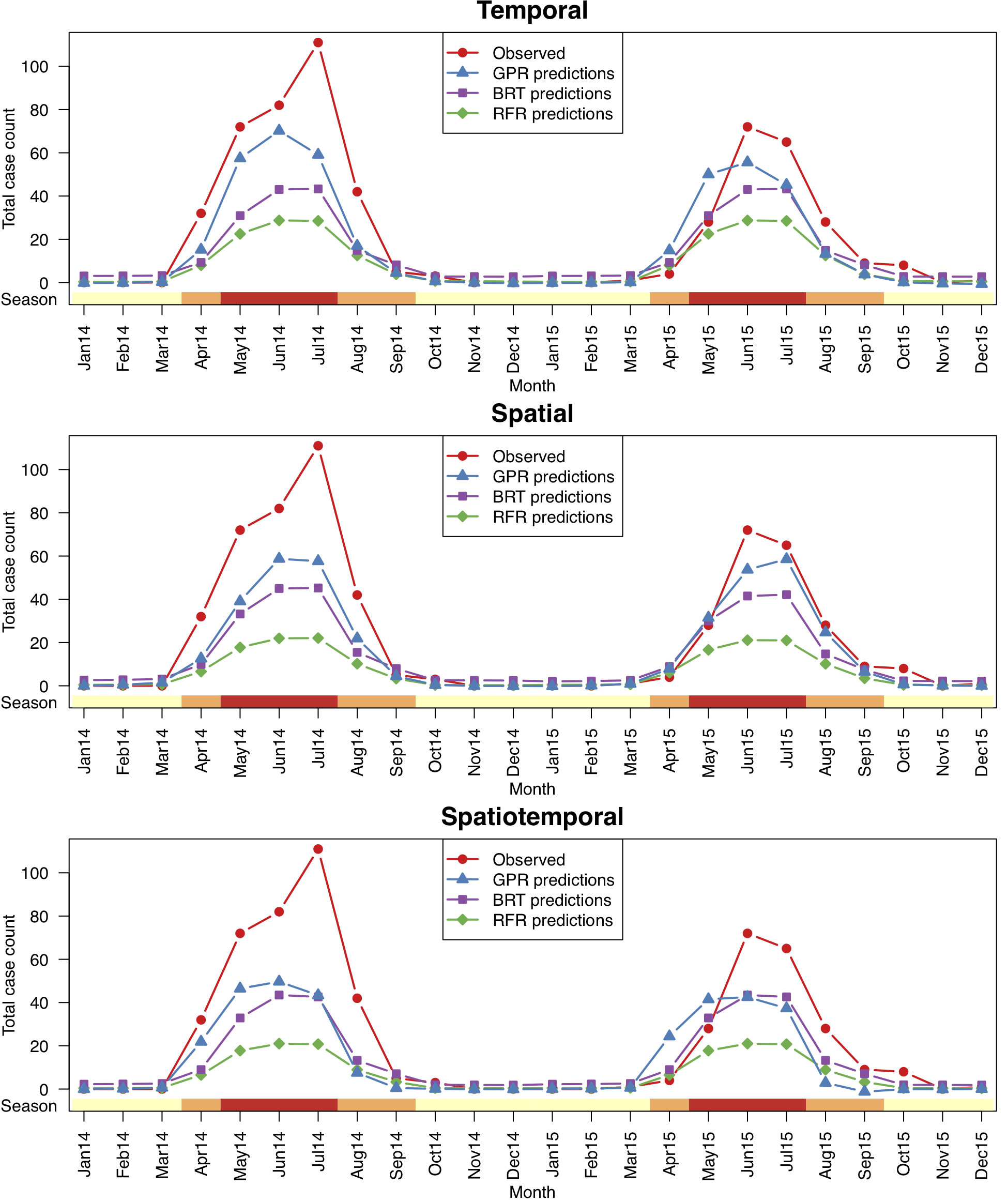

Supplement: S14 Fig — The time periods were annotated by their seasonal group information at the top (yellow: cold; orange: warm; red: hot). (TIFF) [file pntd.0006737.s014.tiff]

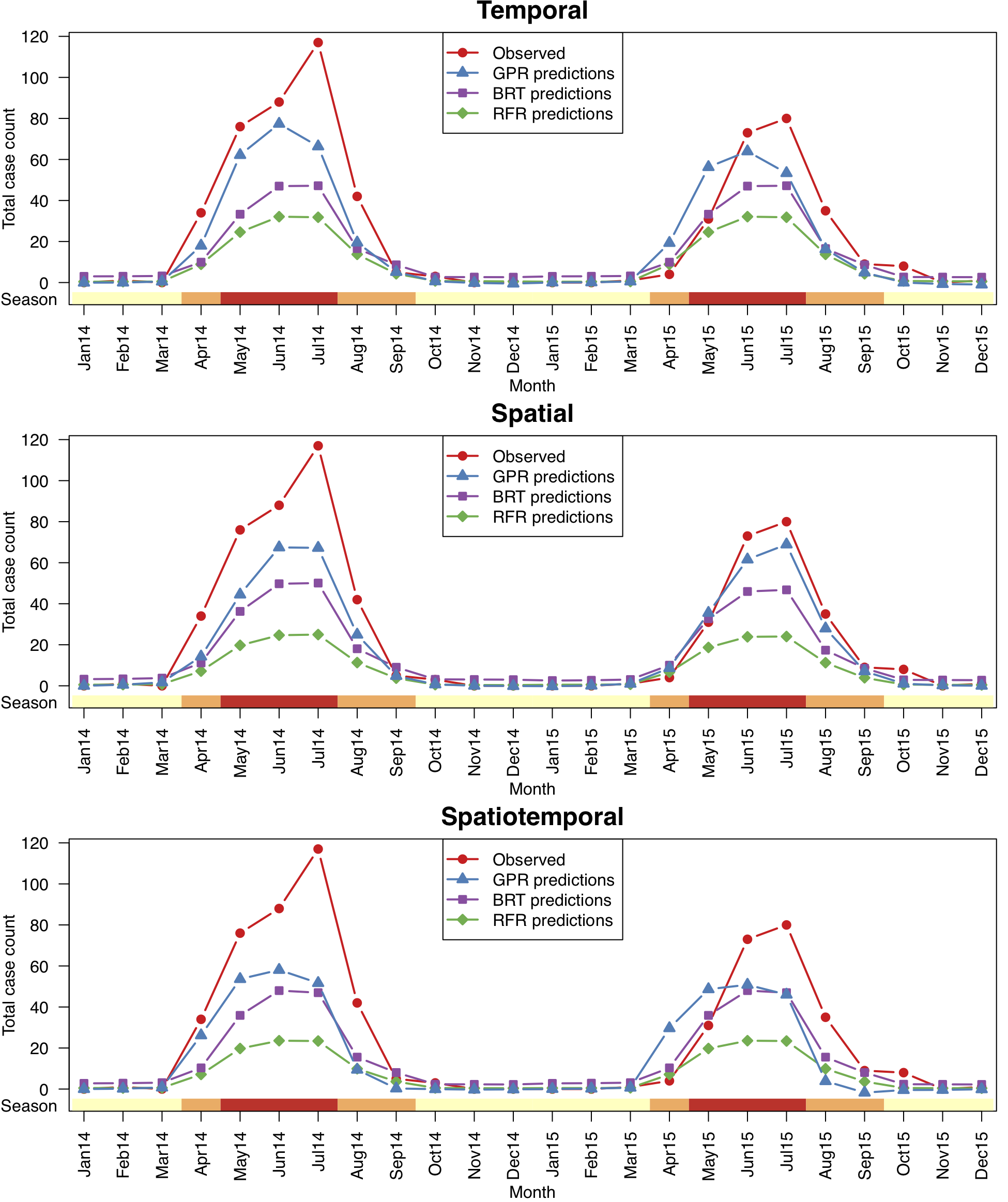

Supplement: S15 Fig — The time periods were annotated by their seasonal group information at the top (yellow: cold; orange: warm; red: hot). (TIFF) [file pntd.0006737.s015.tiff]

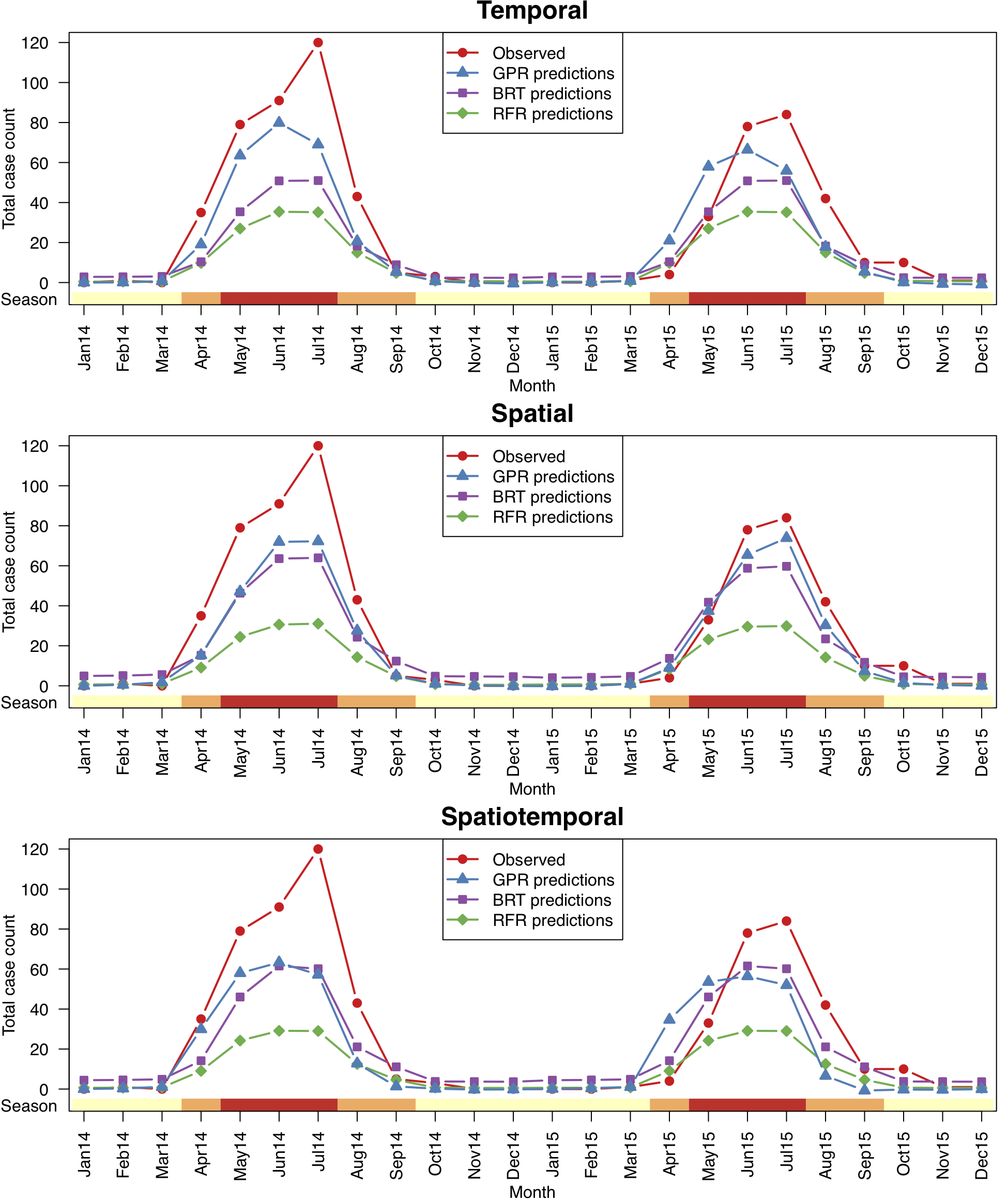

Supplement: S16 Fig — The time periods were annotated by their seasonal group information at the top (yellow: cold; orange: warm; red: hot). (TIFF) [file pntd.0006737.s016.tiff]

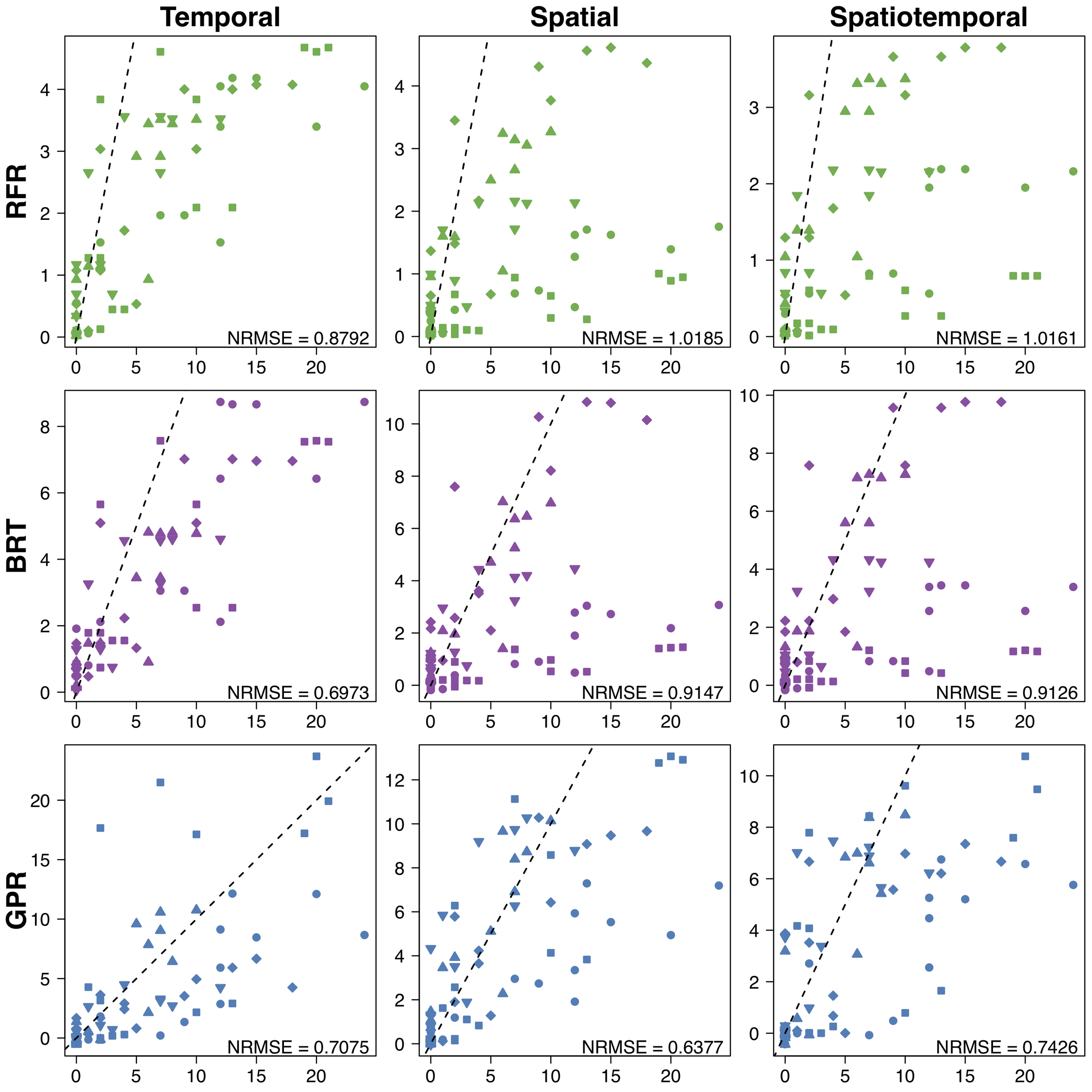

Supplement: S17 Fig — Each province was represented with a distinct marker. We also reported NRMSE values for each algorithm and scenario pair at the bottom-right corner. We also drew a dashed unit slope line to show whether the algorithms captured the range of observed CCHF case counts. Note that BRT and GPR algorithms obtained comparable results for temporal scenario, whereas GPR algorithm achieved remarkably better prediction performances than RFR and BRT algorithms under other two scenarios. (TIFF) [file pntd.0006737.s017.tiff]
